# Sequential and Simultaneous Immunization of Rabbits with HIV-1 Envelope Glycoprotein SOSIP.664 Trimers from Clades A, B and C

**DOI:** 10.1371/journal.ppat.1005864

**Published:** 2016-09-14

**Authors:** P. J. Klasse, Celia C. LaBranche, Thomas J. Ketas, Gabriel Ozorowski, Albert Cupo, Pavel Pugach, Rajesh P. Ringe, Michael Golabek, Marit J. van Gils, Miklos Guttman, Kelly K. Lee, Ian A. Wilson, Salvatore T. Butera, Andrew B. Ward, David C. Montefiori, Rogier W. Sanders, John P. Moore

**Affiliations:** 1 Department of Microbiology and Immunology, Weill Medical College of Cornell University, New York, New York, United States of America; 2 Department of Surgery, Duke University Medical Center, Durham, North Carolina, United States of America; 3 Department of Integrative Structural and Computational Biology, International AIDS Vaccine Initiative (IAVI) Neutralizing Antibody Center and the Collaboration for AIDS Vaccine Discovery (CAVD), The Scripps Research Institute, La Jolla, California, United States of America; 4 Department of Medical Microbiology, Academic Medical Center, University of Amsterdam, Amsterdam, The Netherlands; 5 Department of Medicinal Chemistry, University of Washington, Seattle, Washington, United States of America; 6 Center for HIV/AIDS Vaccine Immunology and Immunogen Discovery, The Scripps Research Institute, La Jolla, California, United States of America; 7 The Skaggs Institute for Chemical Biology, The Scripps Research Institute, La Jolla, California, United States of America; Miller School of Medicine, UNITED STATES

## Abstract

We have investigated the immunogenicity in rabbits of native-like, soluble, recombinant SOSIP.664 trimers based on the *env* genes of four isolates of human immunodeficiency virus type 1 (HIV-1); specifically BG505 (clade A), B41 (clade B), CZA97 (clade C) and DU422 (clade C). The various trimers were delivered either simultaneously (as a mixture of clade A + B trimers) or sequentially over a 73-week period. Autologous, Tier-2 neutralizing antibody (NAb) responses were generated to the clade A and clade B trimers in the bivalent mixture. When delivered as boosting immunogens to rabbits immunized with the clade A and/or clade B trimers, the clade C trimers also generated autologous Tier-2 NAb responses, the CZA97 trimers doing so more strongly and consistently than the DU422 trimers. The clade C trimers also cross-boosted the pre-existing NAb responses to clade A and B trimers. We observed heterologous Tier-2 NAb responses albeit inconsistently, and with limited overall breath. However, cross-neutralization of the clade A BG505.T332N virus was consistently observed in rabbits immunized only with clade B trimers and then boosted with clade C trimers. The autologous NAbs induced by the BG505, B41 and CZA97 trimers predominantly recognized specific holes in the glycan shields of the cognate virus. The shared location of some of these holes may account for the observed cross-boosting effects and the heterologous neutralization of the BG505.T332N virus. These findings will guide the design of further experiments to determine whether and how multiple Env trimers can together induce more broadly neutralizing antibody responses.

## Author Summary

Native-like SOSIP trimers are a platform for development of immunogens aimed at inducing broadly neutralizing antibodies and, hence, a possible vaccine against HIV-1 infection. No previous study has reported on immune responses to more than one such trimer. Here, we assess how rabbits respond to immunization with two or three different trimers, based on virus sequences from HIV-1 clades A, B and C, to gain insights into whether each is immunogenic under various regimens. We find that autologous Tier-2 neutralizing antibody responses can be raised against each trimer immunogen, whether they are delivered simultaneously or sequentially. We also observed some boosting of the neutralization response to the first trimer when a second trimer was administered later. Cross-reactive neutralizing antibodies were seen but only sporadically. We also found that the key immunogenic epitopes on the Env trimers involved holes in the glycan shield, which normally protects the virus from antibody binding. These various findings will guide the design of future experiments in animals and eventually in humans.

## Introduction

Multiple recombinant, soluble envelope glycoprotein (Env) trimers from human immunodeficiency virus type 1 (HIV-1) are currently being produced as immunogens for induction of neutralizing antibody (NAb) responses [1–5]. As NAbs act by recognizing native Env trimers on the HIV-1 surface, trimer-based immunogens intended to induce NAbs should mimic the native structure as closely as possible [6–12]. The SOSIP.664 design of soluble native-like trimers is based on the natural cleavage of the gp140 precursor protein into its gp120 and gp41_ECTO_ subunits and stabilization of the metastable trimer by engineered sequence changes [13]. Multiple native-like SOSIP.664 trimers based on *env* sequences from clades A, B and C have now been described [7, 9, 10, 14–16]. When tested as individual immunogens in rabbits, two such trimers (BG505, clade A; B41, clade B) induced consistently high titers of NAbs against the autologous viruses, which are classified as Tier-2 on the neutralization sensitivity spectrum [5, 16]. While the generation of autologous Tier-2 NAbs is likely to be a necessary step in a vaccine-development program, it is clearly not sufficient [17–20]. The diversity of circulating HIV-1 strains is so extensive that, for immunogens to be practically useful, they must be able to induce broadly active NAbs (bNAbs) that can counter a wide range of viruses. The key unanswered question is how such bNAbs can be induced.

Several approaches to the bNAb-induction problem have been proposed. One strategy involves the use of engineered Env proteins designed to engage bNAb-germline antibodies and provide a path towards affinity maturation [21–27]. Others are based on the use of sequential immunogens derived from infected individuals that developed bNAbs [24, 26, 28–32]. However, one technically straightforward method that requires further evaluation is the simple combination of genetically diverse trimers, such as those based on different clades. The availability and immunogenicity of the clade A BG505 and clade B B41 SOSIP.664 trimers allow appropriate experiments to be devised and conducted in rabbits. Two obvious ways to combine trimers are to deliver them simultaneously or sequentially, and we have assessed both approaches. We then extended the study in time to assess the effect of heterologous boosting with a clade C trimer, either DU422 or CZA97.012, both as SOSIP.664. Overall, the experiment allows us to address several questions relevant to the induction of Tier-2 NAb responses: Do trimers interfere with each other’s immunogenicity or do they generate independent or even reinforcing antibody responses? Does cross boosting occur when a second or third trimer is given to animals previously immunized with a different trimer? Can the use of more than one trimer increase the breadth of the NAb response at the Tier-2 level?

Taken together, our data suggest that the autologous Tier-2 NAb responses to different SOSIP.664 trimers are generated independently, any interference effects being at most modest; that cross boosting can occur; and that while some cross-neutralizing Tier-2 NAbs can arise when more than one trimer is used, they are limited in frequency, breadth and magnitude. The mechanisms underlying several of these observations may be based on observations that the autologous NAb responses to the BG505.T332N, B41 and CZA97 trimers target specific holes in the glycan shields of the corresponding viruses, some of which are shared between immunogens. The inference is that, if trimer cocktails are to be pursued, particularly for boosting responses initiated by germline bNAb-targeting trimers, their composition should not be chosen randomly, but rather based on knowledge of the antigenicity, structure and immunogenicity of each individual component. Moreover, knowledge of how individual trimers induce autologous Tier-2 NAb responses will aid in engineering of improved variants with greater immunogenicity.

## Methods

### Immunogen production

**Fig 1:**
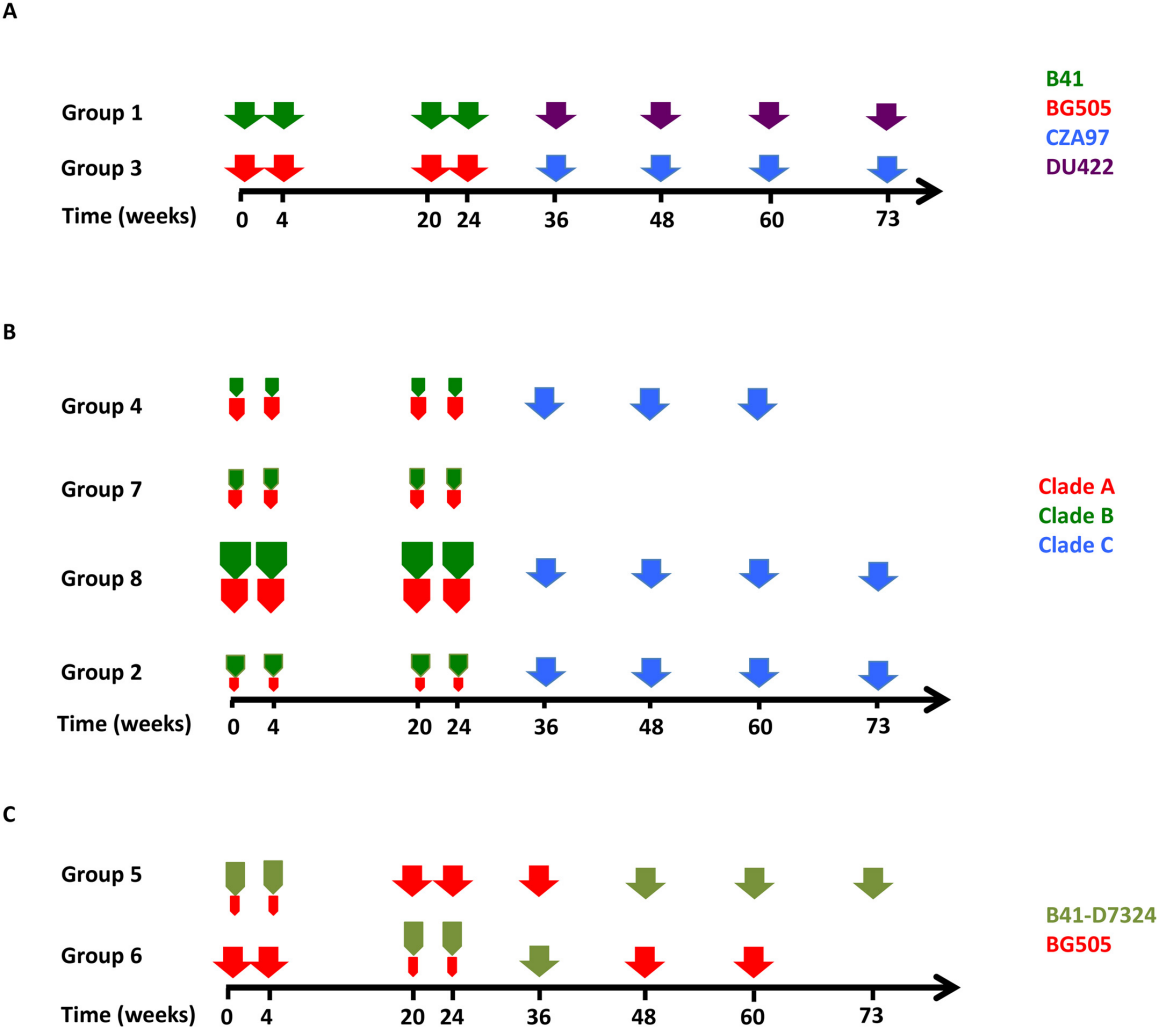
Schematic of the immunization schedule. The longitudinal course of the various sub-studies, in weeks, is depicted, with each immunization indicated by an arrow. Every animal in each group was immunized as indicated at the time-points, except at week 73, when only a subset of animals was immunized (specifically #5715–1, #5716–1, #5718–2, #5719–2, #5721–2, #5726–3, #5727–3, #5734–5, #5735–5, #5736–5, #5749–8). The rabbits were bled to obtain sera for NAb and serology assays immediately before and 2 weeks after each immunization. **A**, The color of the arrows denotes the identity of the trimer, indicated in the key to the right. The amount of trimer given at each time was 30 μg. **B**, The schematic is similar to the one in panel A. The size of the arrows is proportional to the dose of the trimer. For groups 4 and 7, the total trimer dose in the week 0–24 period was 30 μg, whereas in group 8 it was 90 μg, with clade A (BG505)- and clade B (B41)-based trimers present in ~1/1 ratios (see text). For group 2, the clade A (BG505) content of the mixture (total 30 μg) was ~20% (~6 μg). Clade C (CZA97) trimers (30 μg) were used to boost groups 4, 8 and 2 in the period from week 36 to 60, as indicated. Group 7 was terminated at week 26, group 4 at week 62. **C**, The schematic is similar to panel-A, with the color code to the right. The sizes of the symbols approximately correspond to the absolute and relative doses of the trimer immunogens (see text).

The designs and *in vitro* properties of the BG505 and B41 SOSIP.664 trimers have been described previously [10, 15] as well as the D7324 epitope-tagged version of B41 SOSIP.664 trimers, designated B41 SOSIP.664-D7324 (abbreviated to B41-D7324) [15]. All three of these trimers were produced in stably transfected Chinese Hamster Ovary (CHO) cell lines (Flp-in CHO cell line from Thermo-Fisher (Cat# R75807)). Unfortunately, because of a cell line-contamination problem that was not detected until after the first phase of the immunization experiment was completed, the B41-D7324 trimer preparation contained ~25% of co-purified BG505 SOSIP.664 trimers. The suspected contamination was detected by liquid chromatography-mass spectrometry of the immunogen preparation. Briefly, the purified trimer samples were denatured, reduced, alkylated, deglycosylated with PNGaseF, and digested either by trypsin or GluC protease. The trimer composition was estimated from the relative intensities of the peptides unique to each construct [33]. Once the problem was known, the immunizations were continued with contaminant-free B41-D7324 trimers made from a different cell line (see Fig 1). For late boosts, SOSIP.664 trimers derived from two different clade C viruses were used: CZA97.012 (abbreviated to CZA97) transiently expressed in 293F cells (derived from human embryonic kidney 293 cells, as FreeStyle 293-F cells from Thermo-Fisher (Cat# R79007), [34]) and DU422 expressed in a stable CHO cell line [14] (Fig 1). All stable CHO cell lines were made by the same procedures described elsewhere for the BG505 SOSIP.664 line, and have broadly comparable properties [35]. As only the SOSIP.664 trimer design was used in these experiments, that descriptor is usually omitted from hereon.

The BG505, B41, B41-D7324 and DU422 trimers were all purified by 2G12 affinity chromatography followed by size-exclusion chromatography (SEC), while the CZA97 trimers were purified by PGT151 affinity chromatography, all as previously described [10, 14, 15, 34]. Each trimer preparation was highly homogeneous and fully native-like (>95%) when analyzed by gel electrophoresis and negative-stain electron microscopy [10, 14, 15, 34]. All of the immunogen preparations were prospectively or retrospectively verified by liquid chromatography mass spectrometry as described above. The authenticity of all the cell lines was confirmed by specific PCR amplification of a 421-bp segment spanning the gp120-gp41 junction in the respective *env* gene inserts. No other contamination was detected at the cell-line or purified-trimer stages.

BG505-D7324, B41-D7324 and DU422-D7324 trimers were used as ELISA antigens for determining serum antibody titers [10, 14, 15]. The BG505-D7324 and B41-D7324 trimers were expressed in CHO cells, the DU422-D7324 trimers in 293F cells, and each was purified by the same 2G12/SEC method used for non-tagged trimers. CZA97 SOSIP.664-His (CZA97-His) trimers, also used for serology ELISA, were produced in 293F cells and purified by PGT151-affinity chromatography and SEC [34].

### Immunization procedures and ethics statement

This study was approved and carried out in accordance with protocols provided to the Institutional Animal Care and Use Committee (IACUC) at Covance Research Products (CRP) Inc. (Denver, PA), approval number C0014-15. The rabbits were kept, immunized and bled at Covance in compliance with the Animal Welfare Act and other federal statutes and regulations relating to animals, and adhered to the Guide for the Care and Use of Laboratory Animals, National Research Council, 1996.

Rabbit immunizations and blood sampling were carried out under contract at CRP according to the schedule presented in Fig 1, and essentially as described previously (5). Female New Zealand White rabbits (5 per group) were immunized intramuscularly with trimers at various doses (see Results), formulated with 75 Units of Iscomatrix, a saponin-based adjuvant obtained from CSL Ltd. (Parkville, Victoria, Australia) via the International AIDS Vaccine Initiative [36].

### Viruses and neutralization assays

Neutralizing antibodies in rabbit sera were detected and quantified with Env-pseudotyped viruses in the TZM-bl cell assay as described previously (Tzm-bl cells are derived from the HeLa cell line and supplied by the NIH AIDS Reagents Program, Catalog Number 8129 [5, 10, 15]). For additional information on this assay and all supporting protocols see: http://www.hiv.lanl.gov/content/nab-reference-strains/html/home.htm. NAb assays were carried out at either Duke University Medical Center (DUMC) [37, 38] or the Weill Cornell Medical College (WCMC) [5, 15, 37]. Env-pseudotyped viruses used at DUMC were made with the SG3Δenv backbone [39]; at WCMC, the NL-Luc-AM vector was used [40]. For BG505 neutralization, the autologous Env-pseudotyped viruses bore either full-length BG505.T332N (at WCMC) or cytoplasmic-tail-deleted BG505.T332NΔCT (at DUMC) envelope glycoproteins. When the two variants were directly compared no differences in neutralization sensitivity were observed. The CZA97 cl.12 virus (hereafter, CZA97) is autologous to the CZA97 SOSIP.664 trimer. The DU422.K295N.D386N (hereafter, DU422) autologous virus contains the same two glycan-site knock-in mutations at positions 295 and 386 that were incorporated into the DU422 SOSIP.664 trimer immunogen [14]. All three of the B41, CZA97 and DU422 Env-pseudotyped viruses were based on full-length Env glycoproteins. Their Tier-2 classifications, which were determined at DUMC, have been described elsewhere [37, 41].

In addition, sera from a subset of time points were tested at DUMC against two Tier-1A Env-pseudotyped viruses, MN.3 (clade B) and MW965.27 (clade C); and against a panel of nine Tier-2 viruses: Ce703010217_B6 (clade A), 246-F3_C10_2 (clade AC), CNE55 (CRF01_AE), TRO.11 (clade B), X1632-S2-B10 (clade B), BJ0X002000.03.2 (CRF07_BC), CH119.10 (CRF07_BC), 25710–2.43 (clade C) and Ce1176_A3 (clade C) [42, 43]. An amphotropic murine leukemia virus (MLV) Env-pseudotyped virus [44] was used as a negative control at both sites to determine non-specific inhibition of infection by rabbit sera. In each assay, all serum dilutions were tested in duplicate. Neutralization was defined as the reduction (%) of the infectivity obtained in the absence of serum. The serum dilution factors reducing infectivity by 50% were calculated from nonlinear regression fits of a sigmoid function (with maximum constrained to ≤100% and minimum unconstrained) to the normalized inhibition data by the use of Prism software (Graphpad). For convenience, the resulting reciprocal titers or dilution factors [1/(IC_50_ [ppV])] or reciprocal inhibitory dilution [1/(ID_50_ [ppV])] are henceforth referred to simply as “titers” or “IC_50_ values”. We sometimes observed that neutralization curves plateaued at values of <100% (sometimes < 50%), particularly with the B41 Env-pseudotyped virus; a mechanistic analysis of the variable plateaus will be described elsewhere. We recorded IC_50_ values only when the maximum percentage neutralization was > 50%; other values were recorded as IC_50_ < 20. The titers against the MLV control virus were consistently < 30. We therefore deemed titers >40 to represent robust neutralization.

### Mapping autologous NAb responses using Env-pseudotyped virus mutants

Mutant BG505 and B41 full-length *env* genes containing point substitutions, most of which introduced potential sites of N-linked glycosylation, were made as previously described (5, 10, 15). Env-pseudotyped virus mutants based on these genes, together with the corresponding wild-type viruses, were used in TZM-bl cell neutralization assays, as described above. Viruses based on various clones of the MG505 virus are derived from the mother of the BG505 HIV-1-infected infant [45, 46]. The amino-acid sequences of the MG505 cl.A2 and MG505 cl.H3 clones used in this study, and their relationship to the BG505 sequence, are shown in S1 Fig. The CZA97 cl.12 and cl.29 Env-pseudotyped viruses differ at several Env positions, as described in S1C Fig. Mutants of the CZA97 cl.12 virus, which is autologous in sequence to the CZA97 SOSIP.664 trimers, were made as described elsewhere [47]. The infectivities of the various mutant viruses were similar (within 10-fold) to those of the corresponding parental viruses.

The mutant Env-pseudotyped viruses were tested in TZM-bl cell neutralization assays as described above. At a low dilution of serum (1/50 for BG505 and B41 at WCMC, or 1/60 for CZ97 at DUMC), the extent of neutralization was expressed as a percentage of that obtained with the wild type virus (defined as 100%). In addition, sera were titrated to > 40,000-fold dilution, and the inhibition curves for wild-type and mutant viruses were compared.

### Structural modeling of the BG505 trimer

The trimer model was derived from the PDB 5ACO structure [48]. The glycan added at position 241 was modeled in accordance with the homologous glycan in JR-FL Env (PDB 5FUU; [49]) the glycan at 289, for which there are no experimental data, was modeled as a Man_3_ structure. Modeling and visualization were performed by the UCSF Chimera method (PMID 15264254).

### Capture ELISA for anti-Env binding antibody titers

Anti-trimer capture ELISAs with the BG505-D7324, B41-D7324 and DU422-D7324 trimers were performed as described previously [10]. Briefly, the Env protein antigens were captured onto the solid phase by the sheep antibody D7324 (Aalto Bio Reagents, Dublin, Ireland), the rabbit sera were titrated, and the Env-bound antibody detected. The CZA97-His trimers were captured by an anti-His antibody (6x-His Epitope Tag Antibody 4A12E4, Thermo Scientific), but the other steps of the assay were identical to those in the D7324-based ELISA. Midpoint titers (EC_50_) were derived from the titration curves and serve as measurements of the trimer-binding antibody response to the immunogens [10, 15].

### ELISA for antibodies to the D7324-epitope tag on B41-D7324 trimers

The peptide APTKAKRRVVQREKR corresponding to the epitope-tag on the B41-D7324 trimers was obtained from GenScript, as was a scrambled control peptide, KKQARRARPVREVKT). The peptides were used to coat Maxi-Sorp (Nunc) ELISA plate wells at 0.6 μg/ml. After standard washing and blocking procedures, rabbit sera were added at a dilution of 1/20 in PBS with 2% milk and bound antibodies were detected with a goat anti-rabbit IgG-HRP conjugate diluted 1/3000 (BioRad). As positive controls, sheep Ab D7324 was titrated from 10 to 0.013 μg/ml, and a pool of IgG purified from HIV-1 infected humans (HIVIG; supplied by the NIH AIDS Reagents Program, as #3957, lot # 140406–23) was titrated from 1000 to 1.3 μg/ml. The detection antibodies were rabbit anti-sheep IgG-HRP (Thermo) and goat anti-human IgG-HRP (BioRad), respectively.

### Statistics

Groups were compared by two-tailed Mann-Whitney U tests. Two time points for the same rabbits were compared by Wilcoxon matched-pairs test; the matched pairs results are presented only when the pairing was efficient, according to correlation analyses within the test. Otherwise the results of Mann-Whitney U test are given. To determine whether titers were significantly above the cut-off value of a titer of 20, we applied the Wilcoxon signed rank test. Correlations were analyzed non-parametrically as Spearman rank correlations and the concomitant significances were calculated. All statistical analyses were performed in Prism (GraphPad).

Group sizes of 5 for rabbit immunization studies limit the robustness of conclusions from group comparisons. Here, and in earlier papers [5, 16], we show that SOSIP trimer-induced autologous NAb titers in responding rabbits can span a >3-log range (i.e., <100 to >10,000), some rabbits not responding at all [5, 16]. Hence, the ranges of responses in groups often overlap.

## Results

### Summary of protocol

The 8 groups of rabbits and the immunization regimens are summarized in Fig 1. As noted in Methods, after the immunizations had been initiated, we discovered that a stable CHO cell line producing B41-D7324 trimers had become contaminated with cells from a BG505 SOSIP.664 trimer-expressing line. The resulting immunogen preparation used until week 24 therefore contained both B41-D7324 and BG505 trimers in an approximate 4/1 ratio (Fig 1). B41-D7324 trimers used after week 24 were made from a non-contaminated line and verified to be authentic (see Methods). The D7324-tag was not itself immunogenic and its presence did not markedly influence the immunogenicity of the B41 trimers (see S1 Text and S1 Table). The same adjuvant, Iscomatrix at 75 U per dose, was used for all the immunizations, which were given intramuscularly. For monovalent immunizations, a trimer dose of 30 μg was always used. In all the groups, the rabbits were bled immediately before and 2 weeks after each immunization to obtain sera that were used in virus-neutralization and Ab-binding assays. The neutralization assays, performed at two different sites (WCMC and DUMC) used Env-pseudotyped viruses and the TZM-bl cell line (see Methods). The principal endpoints were the autologous Tier-2 NAb responses to the immunogen trimers, but we also quantified heterologous NAb responses against both Tier-1 and Tier-2 viruses. In addition, binding titers of antibodies against the cognate SOSIP.664 trimers were determined in an antigen capture ELISA.

### Single immunizations with clade A and B SOSIP.664 trimers (groups 1 and 3)

**Fig 2:**
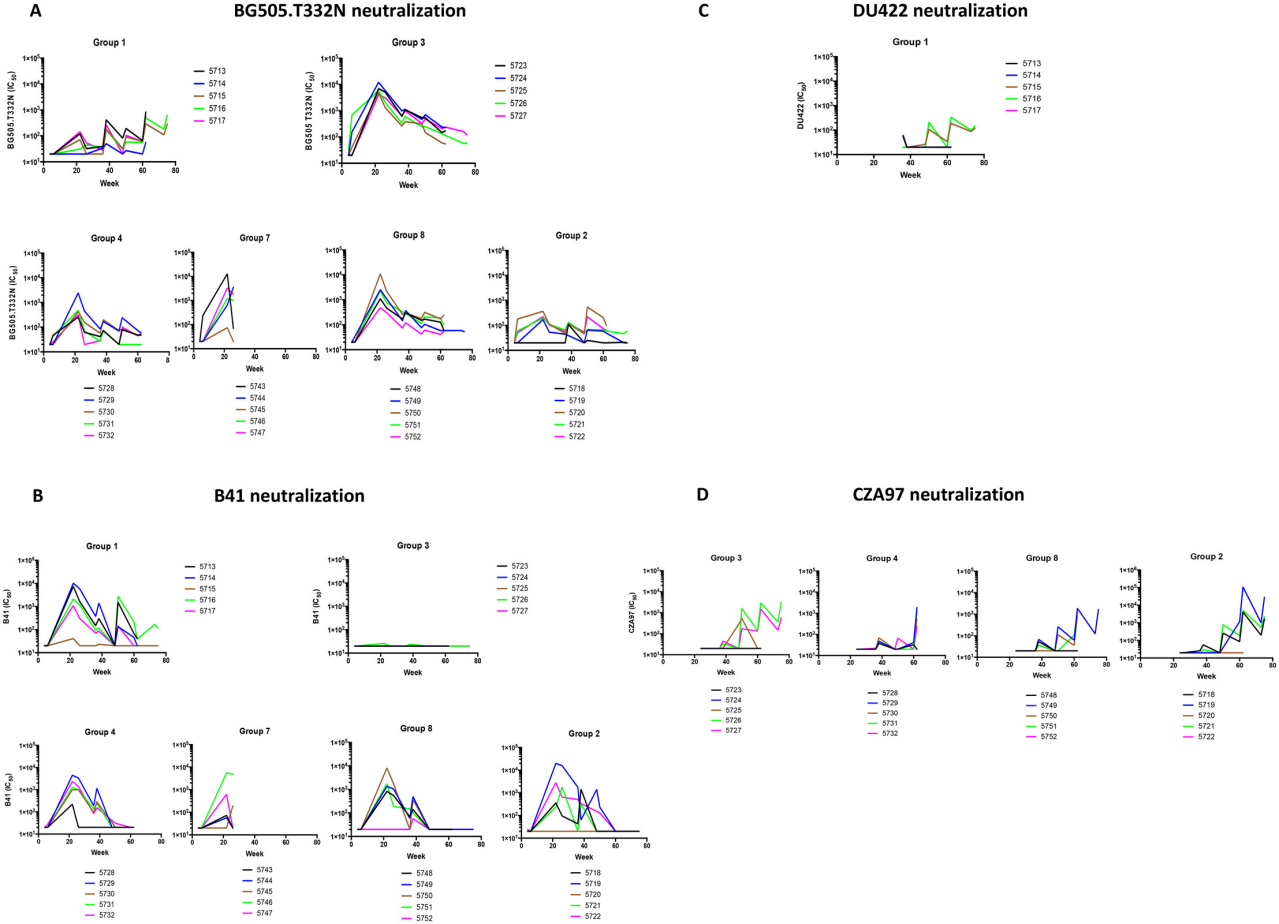
Autologous neutralization responses to clade A, B and C SOSIP.664 trimers. The neutralization titers (IC_50_) against Tier-2 Env-pseudotyped viruses, derived at DUMC, are plotted on the y-axis as a function of time after the first immunization on the x-axis (weeks). The scales are kept constant within each panel to facilitate comparisons among groups. The schedule is shown in Fig 1 and involved immunizations at weeks 0, 4, 20, 24, 36, 48, 60 and 73, with blood taken for analysis immediately before and 2 weeks after each immunization. Note that only a subset of animals was immunized at week 73 (see Fig 1); for the other animals, the final immunization was at week 60. The test viruses were as follows: **A**, BG505.T332N; **B**, B41; **C**, DU422; **D**, CZA97. The curves connecting the reciprocal neutralization titers are color-coded for each rabbit as per the key associated with each group. Thus, changes in titers can be monitored over time both for individual animals and on a group-wide basis.

The initial phases (weeks 0–24) of immunization groups 1 (B41) and 3 (BG505), before heterologous boosting, serve as comparators for other groups (Fig 1A). One BG505 trimer-immunized rabbit (#5726, group 3) developed a strong response after the second immunization (a titer of 680), but that is atypical in our general experience and no such strong early autologous response occurred in the B41 group. Much more consistent autologous NAb responses were raised to each trimer after the third immunization (at week 20) that were poorly boosted by the fourth at week 24 and declined further over the next 12 weeks (Fig 2A and 2B). The autologous NAb responses in the BG505-trimer-immunized group 3 (Fig 2A) at week 22 were in the range 4100–12,000 (median 6000). For group 1 animals that received only the B41 trimers (Fig 1A), the corresponding autologous titer ranges in the four responders were 1100–10,000 (median 4800), while the fifth rabbit (#5715) had a borderline response (peak titer of 40) (Fig 2B). Some moderate cross-neutralization of the BG505.T332N virus occurred at this stage of the experiment (Fig 2A). Three rabbits in the B41 trimer group (#5713, #5715 and #5717) did develop titers >40 (70–140) to BG505.T332N at week 22 (Fig 2A), and other rabbits in this group did so after they were later boosted with clade C trimers (see below). However, the converse was not seen, in that rabbits immunized with BG505 trimers did not develop NAbs against the B41 virus (Fig 2B).

The rest period from the fourth immunization (week 24) to the fifth at week 36 (see below) allowed us to look at how both autologous NAb responses diminished over time. We were unable to determine the half-lives of the titers because of the paucity of time points, and are aware that a second phase of titer decay might commence during the 10-week period from the assay at week 26. However, we note that the autologous responses to the BG505 and B41 trimers in groups 1 and 3 dropped ~10-fold during this time. Responses in these and other groups to later boosting with clade C trimers are discussed further below and are shown in Fig 2C and 2D.

### Single vs. dual simultaneous immunizations with clade A and B trimers (groups 1 and 3 compared to groups 4, 7, 8 and 2)

We analyzed the effect of immunogen dose, and whether one co-administered trimer could interfere with the autologous NAb response to the other. These analyses are described in the SI (see S3 Fig). In summary, we found no strong evidence that either increasing the trimer dose above the 30-μg standard, or the presence of a second trimer, affected the autologous Tier-2 NAb responses to the clade A and B trimers. There was some indication of a modest reduction in the BG505.T332N response at 22 when B41 trimers were co-delivered, but not the converse (S3 Fig). Further studies would be required to determine whether the former outcome is attributable to genuine interference, to a dose-reduction effect, or simply to the random variation associated with a group size of 5 rabbits. The more robust conclusion is that a bivalent mixture of clade A and clade B trimers usually elicited autologous NAb responses to both Tier-2 viruses.

### Sequential immunizations with clade A and clade B trimers

**Fig 3:**
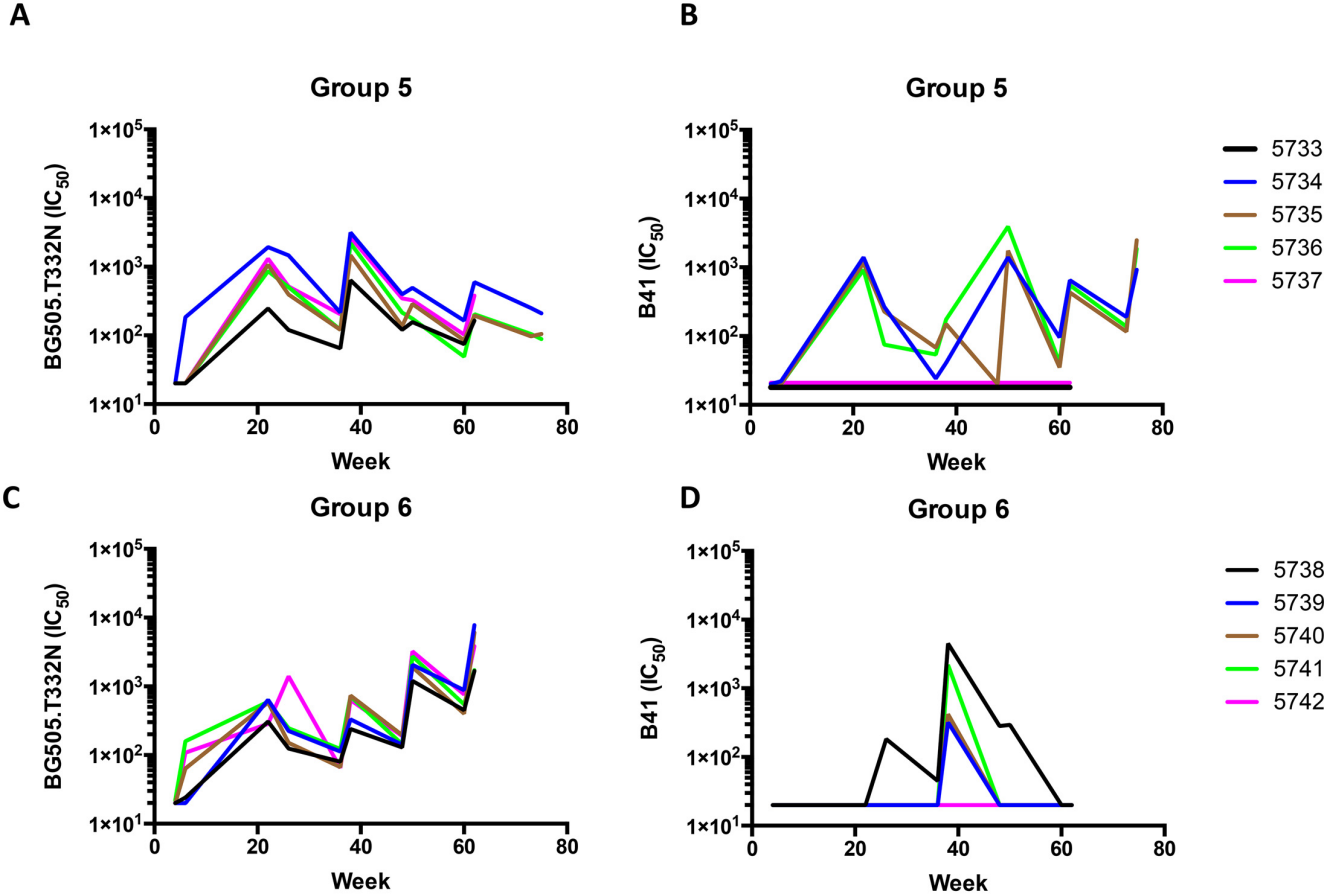
Autologous NAb responses to sequentially delivered clade A and clade B trimers. The neutralization titers (IC_50_) against Tier-2 Env-pseudotyped viruses are plotted on the y-axis as a function of time after the first immunization on the x-axis (weeks). The scales are kept constant within each panel to facilitate comparisons among groups. The schedule is shown in Fig 1C with immunizations at weeks 0, 4, 20, 24, 36, 48 and 60, and for rabbits #5734–5, #5735–5 and #5736–5 also at week 73. Blood was taken for analysis immediately before and 2 weeks after each immunization. The test viruses were as follows: **A** and **C**, BG505.T332N; **B** and **D**, B41. The curves connecting the reciprocal neutralization titers are color-coded for each rabbit as per the key associated with each group. Thus, changes in titers can be monitored over time both for individual animals and on a group-wide basis.

Groups 5 and 6 were also affected by the BG505 contamination (~20%) of the initial B41-D7324 trimer preparations (Fig 1C). Nonetheless, some useful information can be gleaned from the experiment in its full duration (Fig 3). The BG505.T3332N NAb responses for group 5 were quite strong at week 22 (median titer 1100), which may reflect an amplification of responses by the standard BG505 trimer dose (30 μg) at week 20 that were primed by the earlier exposure to lower doses (~6 μg at weeks 0 and 4). The next two standard doses of BG505 trimer at weeks 24 and 36 boosted the BG505.T332N NAb titer only modestly, to a median of 2100 at week 38 (Fig 3A). There was also an increase in the BG505.T332N NAb median titer (from 85 to 200) between weeks 60 and 62, in response to the B41-D7324 trimer boost at week 60 (Fig 3A). This rise could reflect a new cross-reactive response to the clade B trimer or some cross-boosting of the earlier response to the clade A trimer (see below).

The B41 NAb response for group 5 at week 22 was unexpectedly strong in the three responding rabbits, considering that these animals had received only two doses of B41-D7324 trimer (at weeks 0 and 4) and then a heterologous boost by BG505 trimers at week 20 (Fig 3B). Thus, the B41 NAb titers for group 5 at week 22 (median 920) were not significantly lower than those for group 1 (median 2100) at the same time point (p = 0.14), even though the group 1 animals had received the standard three doses of B41 trimers by this time (cf. Fig 2B). Usually, two doses of a trimer are not sufficient to induce a strong autologous NAb response (e.g., see group 1 at week 6, after 2 doses of B41 trimers, Fig 2B). In summary, the effect of the week-20 BG505 trimer immunization on the B41 NAb response of the group-5 rabbits, through cross- boosting, was comparable to that of the third B41 trimer dose on the group 1 rabbits at week 20. We discuss below the possible mechanisms underlying cross-boosting. The B41 NAb titers then declined until they were boosted again by the later B41-D7324 trimer doses at weeks 48 and 60 and, for the three responding rabbits, at week 73 (Fig 3B). Note that two rabbits (#5733 and #5737) did not develop B41 NAbs at any time-point; the autologous NAb epitope(s) on the B41 trimers were not immunogenic in these two animals because of unknown but possibly genetic influences.

The BG505.T332N NAb response in group-6 rabbits was consistently boosted by each immunization given during the 60-week duration of the experiment. This was the case even when B41-D7324 trimers were given at week 36, which is therefore another example of cross- boosting (Fig 3C). Thus, a prolonged trimer immunization regimen involving multiple boosts is feasible. A B41 NAb response in group 6 was seen in 4 of 5 rabbits only after the third exposure to B41-D7324 trimers at week 36, and then declined during the later period of BG505-only immunizations (Fig 3D). This pattern is consistent with that seen with group 1 (B41 trimers only) in the period up to week 22, and from then until the clade C trimer boosts began at week 36 (see Fig 2B). Thus, whereas immunizing with B41 trimers enhanced the BG505.T332N NAb response in both groups 5 and 6, the converse occurred only in group 5 at week 20. Below, we discuss the possible mechanisms underlying these observations.

### Heterologous boosting with clade C SOSIP.664 trimers

We explored heterologous boosting with clade C trimers by immunizing group 1 with DU422 trimers and groups 2, 3, 4 and 8 with CZA97 trimers at weeks 36, 48 and 60, and selected animals also at week 73 (Fig 1A and 1B). In each case, the trimer dose was 30 μg. If we nominally reset week-36 as the 0-time point for this particular study, the rabbits received three immunizations with clade C trimers at weeks 0, 12, 24 and, in some cases, a fourth at week 37. This *ad hoc* extension to the rabbit experiment did not include a study of clade C trimers in naïve rabbits (this experiment is now in progress). Hence, there is no comparator arm to judge whether the response to the DU422 and CZA97 trimers was influenced by prior exposure to the clade A and/or clade B trimers.

After the second immunization at week 48, autologous NAb responses developed in 2 of 5 rabbits immunized with DU422 trimers and were boosted by the third and fourth immunizations at weeks 60 and 73 (titers of 110, 190 and 84 for rabbit #5715, and 210, 350 and 110 for #5716); the other three animals were non-responders but #5713, #5714 and the responder #5715 had cross-reactive titers of 60 at week 36 (Fig 2C). The CZA97 trimers induced more consistent and higher-titer autologous responses, particularly after the third immunization. Overall, autologous NAb titers >100 were seen in 12 of the 20 CZA97 trimer recipients at the week-62 time-point (Fig 2D). The median titer for these 12 responders was 1700, and it was 580 for the entire set of 20 animals. We note that the responses to each of the clade C trimers after the second immunization were more frequent than we generally observe (here and in other studies) to two immunizations with other trimers (see, for example, the week-6 responses to clade A and clade B trimers; Fig 2A and 2B). However, there are too many variables to determine what this means: the trimer genotype, the prior exposure to other trimers, and the spacing between the first and second immunizations (4 *vs*. 12 weeks) could all have influenced the observed outcome.

**Fig 4:**
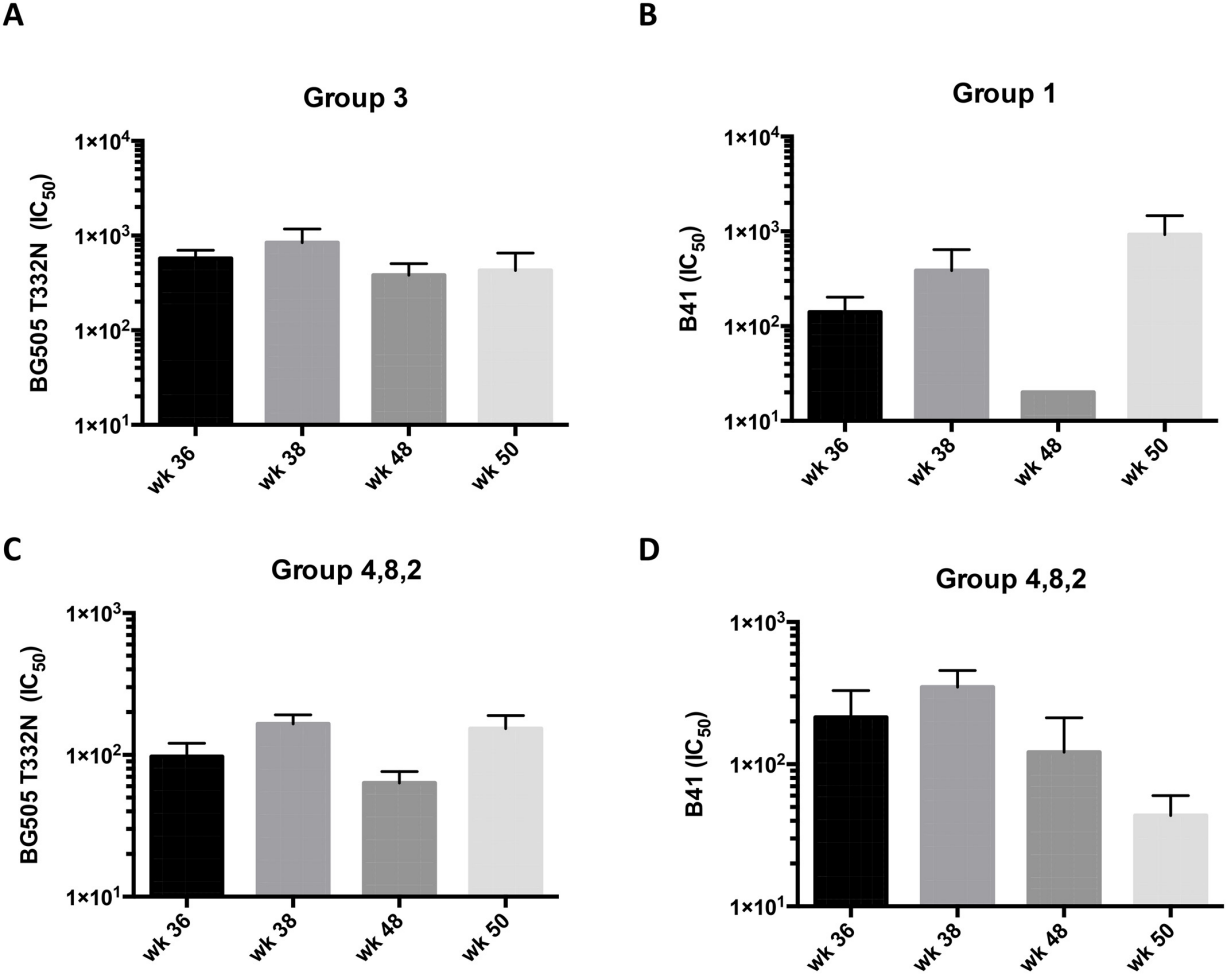
Cross-boosting of clade A and clade B autologous NAb responses by clade C trimers. NAb titers at the indicated time points for all five animals in the listed groups are compared. Bars show arithmetic means + s.e.m. The clade C trimer boosts occurred at weeks 36 and 48. **A** and **C**, BG505.T332N titers; **B** and **D**, B41 titers.

**Fig 5:**
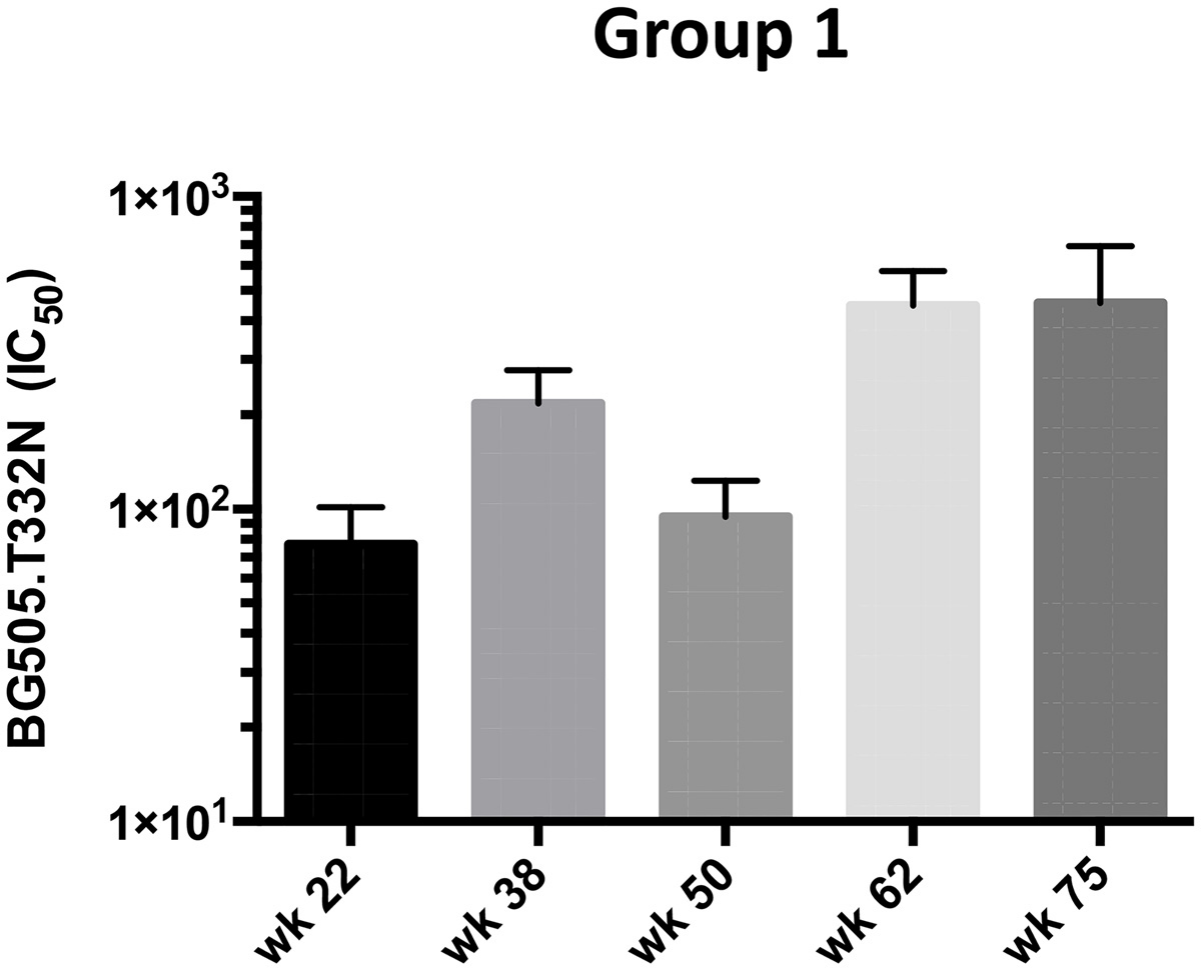
*De novo* cross-neutralization of BG505.T332N (clade A) induced by clade C trimers after priming with clade B trimers. BG505.T332N NAb titers at the indicated time points for the five animals in group 1, which received only clade B and C trimers are shown. Bars show arithmetic means + s.e.m. The NAb titers were compared with the cut-off value of 20 by the Wilcoxon signed rank test (see Results).

The study design allowed us to further investigate whether heterologous trimers from a different clade can cross-boost existing autologous NAb responses. The first immunization (week 36) with clade C trimers (DU422 for group 1, otherwise CZA97) of rabbits previously administered clade A and/or B trimers cross-boosted the existing B41 (groups 1, 2, 4 and 8) and BG505.T332N (groups 3, 2, 4 and 8) NAb titers (measured 2 weeks post-boost, i.e., at week 38) in most animals, although to varying degrees (Fig 2A and 2B). For BG505.T332N neutralization, the titers for group 3 were only marginally higher at week 38 than at week 36 (median 1000 *vs*. 620) and likewise they were higher after the second clade C trimer boost at week 50 than at week 48 (490 *vs*. 330) (Fig 4A). The corresponding effect of the clade C (DU422) boost on the B41 NAb titers for group 1 was also weak for week 38 *vs*. week 36, median 110 *vs*. 91; but significant for week 50 *vs*. week 48, median 140 *vs*. 20; p = 0.048 (Fig 4B). The clade C trimer boosts to groups 2, 4 and 8 combined, which had previously received clade A and B immunogens simultaneously, also cross-boosted higher BG505.T332N NAb titers at week 38 compared with the pre-boost level at week 36 (120 *vs*. 58, p = 0.022); the same effect was seen at week 50 compared with week 48 (p = 0.013) (Fig 4C). In these groups, the B41 NAb titers rose only at week 38 compared with week 36 (medians 240 and 70, p = 0.066); they were not sustained between weeks 50 and 62 (Fig 4D). No cross boosting of the B41 response was seen after week 48. The diminishing effect of cross boosting over time may reflect the increasing period after the last exposure to the initial immunogen(s), i.e. at week 24. The week 60 and 73 immunizations with clade C trimers boosted the BG505.T332N NAb titers for group 1 only, which was a completely heterologous response (no exposure to BG505 trimers) that is described more fully below (Fig 5). Overall, the evidence suggests that immunization with a clade C trimer was able to cross-boost the autologous NAb responses induced by prior exposure to the BG505 trimer or the B41 trimer.

We also explored whether boosting with clade C trimers broadened the overall neutralization response. One notable observation was that NAbs against the BG505.T332N virus were detected in 5 of 5 rabbits from group 1 at week 38 (median titer 180, p = 0.062) (Figs 2A and 5). These animals had been sequentially immunized with B41 and then DU422 trimers, but had never received BG505 trimers (Fig 1A). Hence, they developed a heterologous response to the BG505.T332N virus. The response was even stronger after the week-60 boost (median titer of 530 at week 62, p = 0.062), and the two rabbits (#5715–1, #5716–1) that received a further DU422 trimer immunization at week 73 had BG505.T332N titers of 290 and 640 at week 75 (Figs 2A and 5). Conversely, no NAbs against B41 were induced in the group 3 rabbits that were initially immunized with the BG505 trimer and then boosted with its clade C CZA97 counterpart (Fig 2B).

### Rabbits can develop autologous NAbs to multiple trimers

During the 60- or 73-week immunization period, the 15 rabbits in groups 2, 4 and 8 received three different trimers from clades A, B and C (Fig 1B). All 15 rabbits raised autologous NAb responses (titer >100) to at least one of the trimer immunogens at various time points. Ten of the rabbits were triple responders, in that they raised moderate/strong NAb responses (titers >100) to each of the corresponding autologous viruses (Fig 2A, 2B and 2D; Table 1). Sera from three double responders ((#5728–4, #5748–8 and #5722–2) neutralized the BG505.T332N and B41 viruses but not CZA97, while sera from two rabbits (#5752–8 and #5720–2) neutralized only BG505.T332N (Fig 2A, 2B and 2D; Table 1).

**Table 1:** Individual rabbits immunized with three different trimers can develop moderate to strong autologous NAb responses to each of them^a^.

Rabbit ID-group	BG505.T332N virus		B41 virus		CZA97 virus	
	Max. titer	Week	Max. titer	Week	Max. titer	Week
**Triple Responders**						
5729–4	2500	22	4600	22	1700	62
5730–4	440	22	980	22	480	62
5731–4	490	22	1300	22	1600	62
5732–4	300	22	2300	22	250	62
5749–8	2600	22	1300	26	2100	62
5750–8	11,000	22	7300	22	900	62
5751–8	2400	22	1800	22	680	62
5718–2	130	38	1600	38	3700	62
5719–2	180	22	21,000	22	>44,000	62
5721–2	200	22	1800	26	5300	62
**Double Responders**						
5728–4	260	22	220	22	<100	-
5748–8	1100	22	870	26	<100	-
5722–2	240	22	2800	22	<100	-
**Single Responders**						
5752–8	500	22	<100	-	<100	-
5720–2	550	50	240	-	<100	-

^***a***^ The highest recorded autologous NAb responses to the viruses corresponding to the BG505, B41 and CZA97 trimers are listed, and the time point at which they were measured. The rows are grouped by whether the animals developed moderate/strong titers (>100) to three, two or one trimer.

In groups 5 and 6, after the sequential immunization with BG505 and B41 trimers, all 10 rabbits developed medium/strong NAb responses against BG505.T332N (peak titers 630–8200). Six of these rabbits also raised NAbs against B41 (peak titers 340–4700), whereas the other four (#5733–5 and #5737–5; #5740–6 and #5742–6) did not (Fig 3).

### No correlation between the Tier-1 and autologous Tier-2 NAb responses of individual rabbits to different trimers

**Fig 6:**
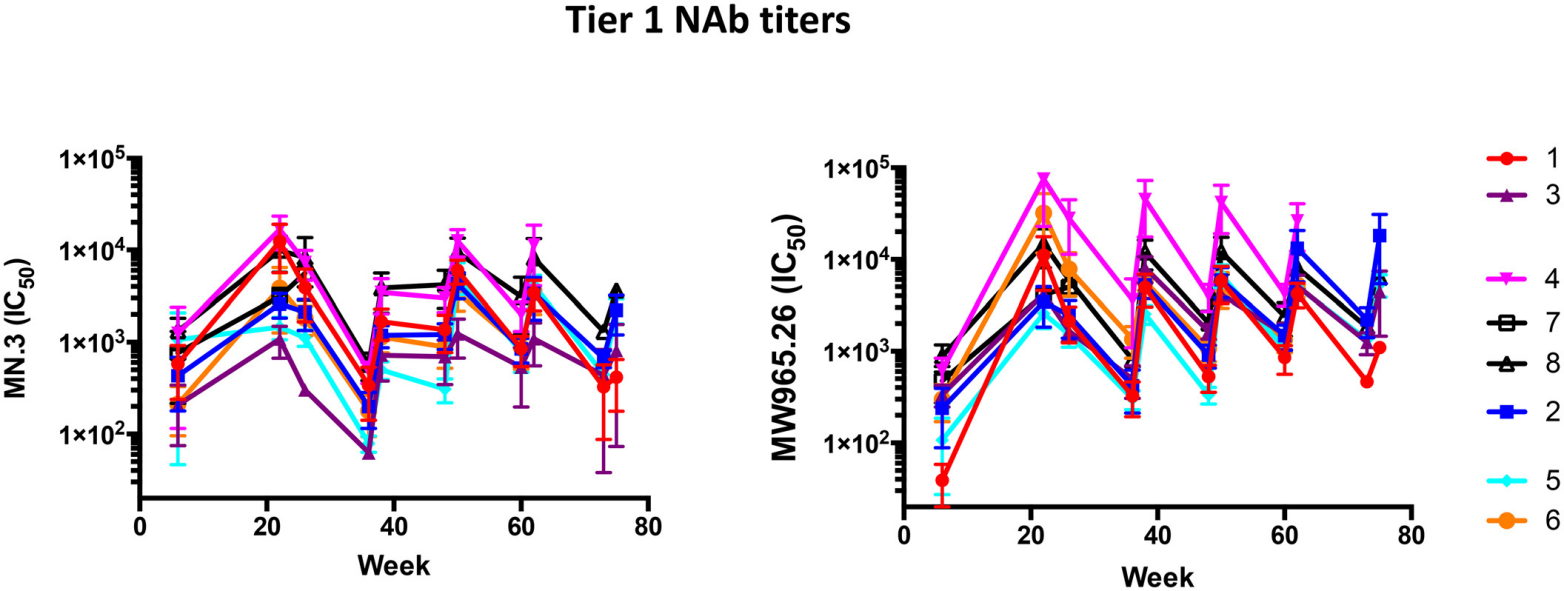
Neutralization of heterologous Tier-1 viruses. The arithmetic mean neutralization titers (IC_50_) against the Env-pseudotyped Tier-1 viruses MN.3 (clade B) and MW965.26 (clade C), derived at DUMC, are plotted over time for the rabbit groups denoted by the key to the right.

We then analyzed the heterologous Tier-1 (MN, clade B and MW965.26, clade C) NAb titers in all rabbits (Fig 6). We compared the Tier-1 NAb titers of “complete responder” rabbits with animals that responded less consistently. The “complete responder” subset included rabbits from groups 2, 4 and 8 that developed autologous Tier-2 NAbs in response to all three trimers they received (n = 10), as well as rabbits from groups 5 and 6 that did so in response to both trimers they were given (n = 6) (see Table 1). The Tier-1 NAb responses of these 16 “complete responder” rabbits (median titers of 1400 against MN.3 and 6200 against MW965.26) were indistinguishable from those of the remaining 9 rabbits from the same groups that developed Tier-2 NAbs less consistently (median titers of 980 against MN.3 and 6200 against MW965.26; p = 0.64 for MN.3 and p = 0.89 for MW965.26). Thus, the Tier-2 autologous and Tier-1 NAb responses induced by the various trimers do not track with one another.

To supplement the above analyses, we plotted the Tier-1 NAb titers (again MN.3 and MW965.26) for all groups against the various autologous Tier-2 NAb titers at weeks 22 and 62 (Fig 6, S2 Fig, S2 Table). Spearman correlation analyses again showed that the Tier-1 and autologous Tier-2 NAb titers were consistently non-correlated. This conclusion is concordant with our previous findings, where we also showed that peptide-reactive, anti-V3 antibodies dominate the Tier-1 NAb response to BG505 SOSIP.664 trimers, and that these responses do not correlate with BG505 neutralization [5].

Why are the autologous Tier-2 NAb responses to multiple trimers strong and consistent in some rabbits, but weak or absent in others? As described above, the poor Tier-2 NAb responses in the latter subset of rabbits are not attributable to a global inability to respond to the trimers, as the Tier-1 NAb titers in these animals were not atypical. The explanation might be rooted in whether the antibody repertoires of individual animals can recognize the types of Tier-2 NAb epitopes that are described below. Thus, whereas all the rabbits may be capable of raising antibodies to Tier-1 NAb epitopes, some may be incapable of responding to Tier-2 NAb epitopes. Moreover, the different characteristics of the epitopes for Tier-1 and Tier-2 NAbs may explain why their titers are uncorrelated.

### Autologous NAb responses target glycan holes

**Fig 7:**
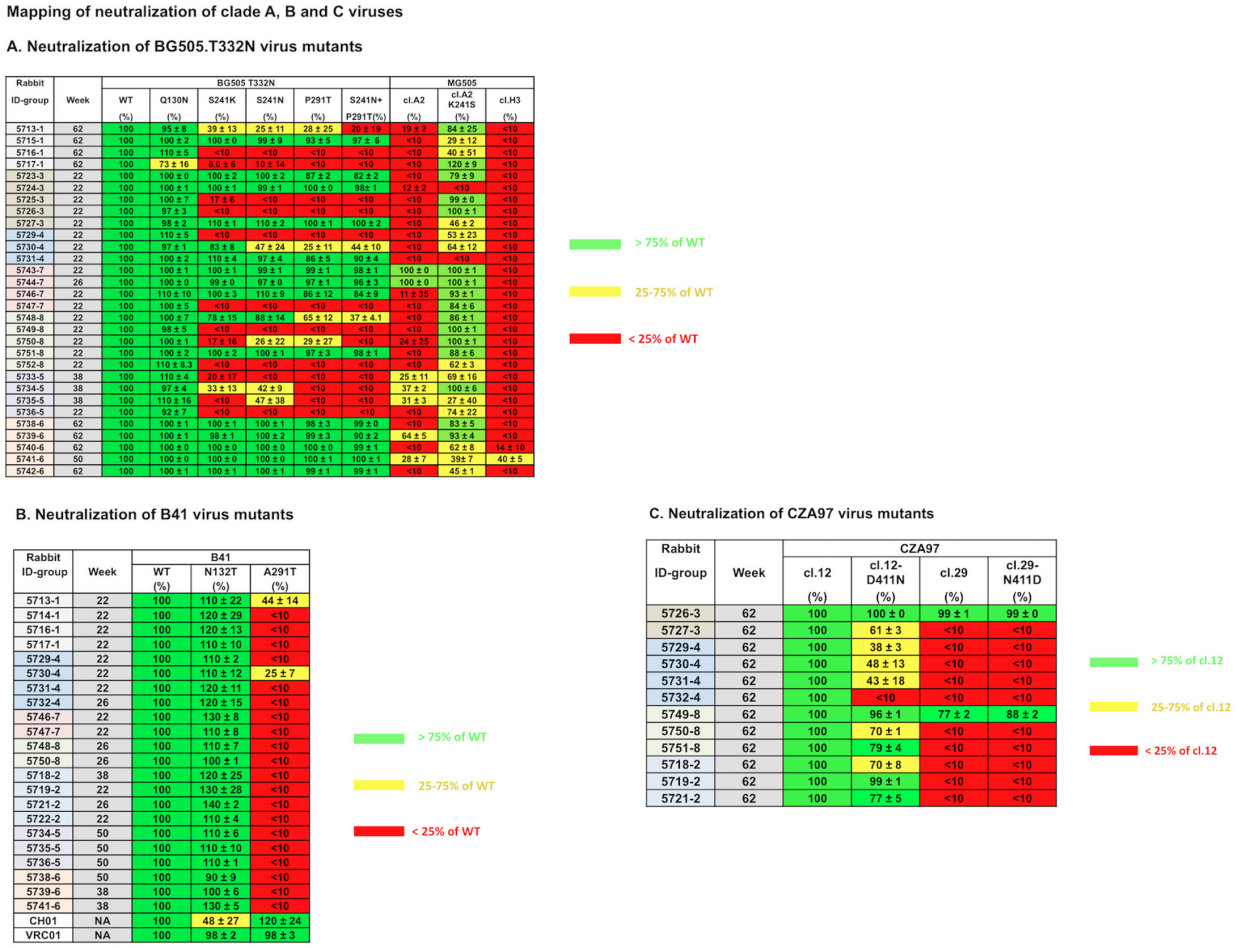
Mapping autologous NAb specificities using BG505.T332N, B41 and CZA97 virus mutants. **A**, Neutralization of BG505.T332N virus mutants. The values recorded for the various mutant viruses are the percentage neutralization at a dilution of 1/50, relative to the BG505.T332N parental virus (labeled WT and defined as 100%), and are the averages of 2 replicates ± s.e.m. Red boxes highlight fully or substantially resistant viruses (<25% neutralization); yellow, moderately resistant viruses (25–75% neutralization); green, sensitive viruses (>75% neutralization). The Q130N, S241N and P291T changes introduce N-linked glycans at positions 130, 241 and 289, respectively. The S241N+P291T double mutant contains glycans at both positions 241 and 289. The MG505 cl.A2 and cl.H3 viruses differ from BG505.T332N at several positions (see S1 Fig). Of note is that MG505 cl.A2 has a lysine residue at position-241 (i.e., as per the S241K mutant), whereas cl.H3 has a glycan site (i.e., as per the S241N mutant). A glycan is present at position-289 in both MG505 clones. The K241S change in MG505 cl.A2 restores the Ser residue that is present at position-241 in the BG505.T332N virus. Full titration curves for the sera showed only a single example of reduced neutralization of the glycan knock-in mutants, compared with BG505.T332N, that was not evident at the standard test dilution of 1/50 (rabbit #5739 at week 62; S4 Fig). Overall, the extent of neutralization of the glycan knock-in mutants did not correlate well with the titers against the wild-type BG505.T332N virus (Spearman rank correlation for the 241-glycan knock-in: r = 0.30, p = 0.099; for the 289-glycan knock-in: r = 0.21, p = 0.25; for the double mutant: r = 0.27, p = 0.15). **B**, Neutralization of B41 virus mutants. The organization is the same as in panel-A. The B41 parental virus is labeled WT and its neutralization defined as 100%. The N132T and A291T changes into this virus introduce N-linked glycans at positions 130 and 289, respectively. The CH01 and VRC01 bNAbs were used as control reagents for assessing overall neutralization sensitivity (only shown for B41 mutants for which a partial effect on CH01 neutralization was observed). **C**, Neutralization of CZA97 virus mutants. The organization is the same as in panel-A. The serum dilution used was 1/60. The CZA97 cl.12 parental virus is labeled cl.12 and its neutralization defined as 100%. The CZA97 cl.29 virus differs from cl.12 at several positions (see S1 Fig) but of note is that it contains a glycan at position-411. The CZA97 cl.12-D411N and cl.29-N411D mutants contain and lack glycans at position-411, respectively.

We based our mapping strategy on the hypothesis that autologous Tier-2 NAbs recognize small holes in the glycan shield where a glycan is absent that is present in multiple HIV-1 strains [50]. Accordingly, we inserted N-linked glycans at specific sites on the BG505.T332N and B41 Env-pseudotyped viruses to fill "glycan holes": For BG505.T332N, these holes are at positions 130, 241 and 289; the resulting BG505.T332N virus mutants are designated BG505-Q130N, BG505-S241N, BG505-P291T (inserting a glycan at position-289) and BG505-S241N+P291T (double mutant containing glycans at positions-241 and -289). We also used clones of the maternal virus MG505, including the cl.A2-K241S mutant in which the lysine in the mother’s virus at residue-241 was changed to the serine found in the infant’s virus [46] (S1 Fig). The converse substitution at residue 241 was used to make the BG505-S241K mutant. For mapping the B41 response, we identified potential glycan holes at positions 130 and 289 by inspecting the sequence in the structural context, and made the B41-N132T (glycan at position-130) and B41-A291T (glycan at position-289) mutants. For CZA97, we used two clones that differ by the presence (cl.29) or absence (cl.12) of a glycan at position-411, together with other nearby substitutions (see Methods and S1 Fig). The neutralization sensitivities of the various viruses to sera from BG505, B41 or CZA97 trimer-immunized rabbits were then assessed in the TZM-bl cell assay (Fig 7).

### Mapping of BG505.T332N neutralization epitopes

We analyzed the autologous NAb response to the BG505 trimer in 30 different rabbits. The key results are based on the peak BG505.T332N responses recorded in each rabbit, and represent relative neutralization at a single serum dilution of 1/50 (Fig 7A). One serum that fully neutralized the mutants at the 1/50 dilution did so with reduced efficacy at higher dilutions when the samples were titrated; in those cases, the relative neutralization titers are given in S4 Fig and reinforce the conclusions drawn below. Additional analyses include how the specificity of the BG505.T332N NAb response changed over time in some rabbits, and whether the immunization regimen influenced the specificity of the response (S1 Text, S3A Table).

**Fig 8:**
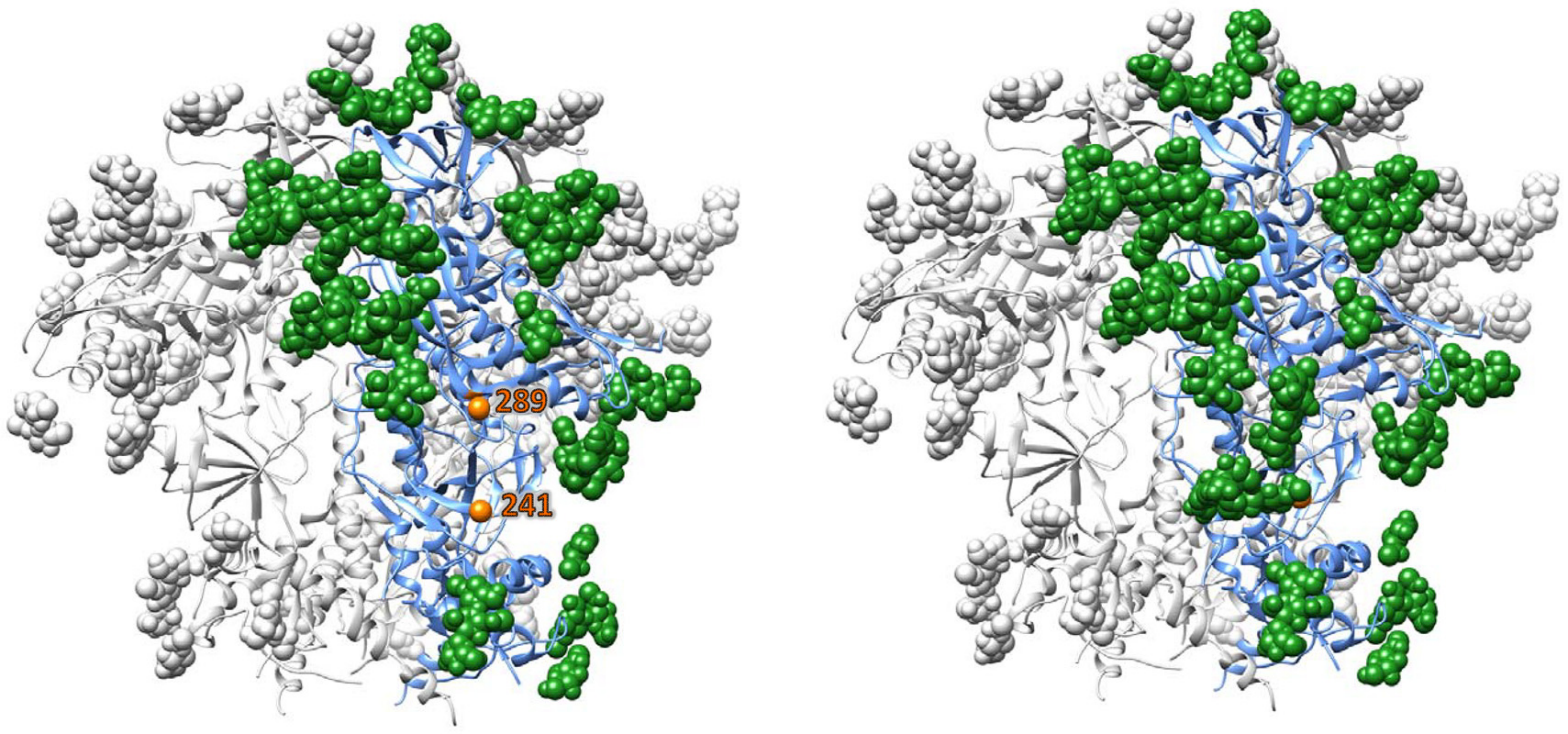
A glycan hole in the BG505 trimer. Left panel: The BG505 SOSIP.664 trimer and the BG505.T332N virus lack glycans at positions 241 and 289, which results in a large exposed peptide surface on the side of gp120 near the gp120/gp41 interface. For clarity one protomer of the BG505 SOSIP.664 trimer has been colored, with the polypeptide chain in blue ribbons and glycans denoted by green spheres. Right panel: The restored glycans at positions 241 and 289 (as in the BG505 S241N+P291T double mutant virus) now mask the underlying peptide surface. The individual glycans at positions 241 and 289 both have a substantial masking effect, accounting for the similar phenotypes of the single and double mutant knock-in viruses (S3 Table).

Overall, the 30 rabbits fell into two major sub-groups based on whether the sera could neutralize BG505.T332N virus mutants containing glycan knock-ins at positions 241 and/or 289, or the S241K point substitution. Neutralization by 16 of the 30 sera was partially or completely eliminated when a glycan was present at both of these sites; the single glycan knock-in mutants and the double 241+289 mutant viruses yielded highly concordant data (Fig 7A). In addition, reduced capacities to neutralize the glycan knock-in mutants were revealed when serum #5739–6 from week 62 was titrated (S4 Fig). Our interpretation, supported by structural modeling, is that each of the knocked-in 241 and 289 glycans occludes the target epitope for prominent autologous NAb specificities present in these 17 sera (Fig 8). In general, the neutralization properties of the S241K mutant tracked those of the glycan knock-in mutants, and indicate that Ser-241 has an important influence on this NAb epitope (Fig 7A).

In contrast, the other 13 sera efficiently neutralized the 241 and 289 single and double glycan knock-in mutant viruses, and also the S241K mutant (Fig 7A, S3A Table). It is possible that these sera target the same region of the trimer in a way that is unaffected by the knocked-in glycans or the presence of a Lys at residue-241. Alternatively, they may target an entirely different and as yet unknown epitope(s) elsewhere on the trimer, in addition to or instead of one located in the 241/289 region. Those epitopes do not involve a glycan hole present on the BG505.T332N virus at residue-130 because 29 of the 30 sera neutralized the Q130N glycan knock-in mutant with the same efficacy as the wild-type virus, and the decrease in neutralization seen with the other serum (#5717–1) was only modest (Fig 7A).

Additional clues, and also complexities, are provided by neutralization assays with clones of the MG505 maternal virus and mutants thereof. These clones differ from BG505.T332N in several positions, including at position-241 (S1 Fig). The MG505 cl.A2 virus was partially or completely resistant to 28 of the 30 sera but, in 26 of those 28 cases, it became more sensitive when the K241S change was made to restore the residue found at this position in the BG505.T332N virus (Fig 7A, S3A Table). The latter effect is just as expected for the sera that fail to neutralize the BG505-S241K mutant. However, we noted four examples (#5723–2, #5746–7, #5751–8 and #5738–6) where the presence of a lysine at position-241 was associated with neutralization resistance in the MG505 context (i.e., compare MG505 cl.A2 with MG505 cl.A2-K241S), but not in the BG505 context (compare BG505.T332N with BG505-S241K, and also the BG505 glycan knock-in mutants with the same phenotype). In those four cases, a lysine at position-241 apparently acts in concert with other sequence differences between the MG505 cl.A2 and BG505.T332N viruses to impair crucial epitopes. Overall, the data suggest that residue-241 has a direct or indirect influence on the NAb epitope recognized by the above four sera, in addition to the more clear-cut role it plays for 17 more of the 30 sera (Fig 7A, S3A Table, S4 Fig).

The MG505 cl.H3 virus was resistant to all 30 of the rabbit sera (Fig 7A, S3A Table, S4 Fig), and has a glycan at position-241 (S1 Fig). Taken together with the data on the cl.A2 viruses, it is possible that the 241/289-region of the BG505 trimer may be targeted more frequently than the studies on the BG505.T332N mutants suggest (i.e., by >17/30 sera). However, the wider sequence context of the MG505 clones presumably plays a role. For example, changes elsewhere may indirectly affect how the epitope(s) in the 241/289 region is presented on MG505 viruses. Alternatively, if other, unknown NAb epitopes on the BG505.T332N virus are also targeted by 13 of the rabbit sera and they are absent on the MG505 viruses, this would increase the impact of changes at residue-241 in the MG505 context (e.g., the K241S change to MG505 cl.A2).

As noted above, heterologous NAbs against BG505.T332N were induced in four group 1 rabbits immunized with B41 followed by DU422 trimers (Figs 2A and 5). These animals were never given BG505 trimers (Fig 1A). When the peak sera from these animals were tested against the BG505 mutant viruses, three of them (#5713, #5716 and #5717) were found not to neutralize the 241/289 double glycan knock-in mutant (Fig 7). This finding suggests that the cross-reactive NAbs target the 241/289 region of the trimer. We note that the B41 trimer has a glycan at position-241 but not -289, while the DU422 trimer has glycans at both positions. In two rabbits (#5713 and #5715), the extents to which the glycan knock-in mutants were neutralized increased over the course of the DU422 trimer immunizations (S3A Table), possibly reflecting the shielding effect of the glycans in this region of the immunogen.

### Mapping of B41 neutralization epitopes

Sera from B41 trimer-immunized rabbits were tested against two virus mutants containing knocked-in glycans to occlude potential holes at positions 130 and 289 (Fig 7B, S3B Table). The B41-N132T variant retained the wild-type neutralization sensitivity to all sera. In marked contrast, the B41-A291T mutant (which has an added glycan at position-289) was completely (<10% neutralization relative to WT) resistant to neutralization by 20 of the 22 sera and partially resistant to the other two (Fig 7B). Thus, the autologous NAbs induced by the B41 trimer predominantly target a hole in the shield caused by the absence of a glycan at position-289. The finer details of this epitope remain to be explored by the use of additional mutant viruses.

### Mapping of CZA97 neutralization epitopes

We tested two clones of the CZA97 virus and found that they were either highly sensitive (cl.12; 12 of 12 rabbits) or strongly resistant (cl.29; 10 of 12 rabbits) to sera from rabbits immunized with CZA97 trimers (Fig 7C). A notable difference between these two clones is the presence of a glycan at position-411 in the V4 region of cl.29 that is absent from cl.12, together with 7 single-residue changes between residues 388 and 415 (see alignment in S1 Fig). Thus, we hypothesized that the autologous NAb response to the CZA97 trimers targets the glycan hole at position-411. Accordingly, we made the CZA97 cl.12-D411N and cl.29-N411D point-mutant viruses, respectively, to introduce and delete a glycan at this position in the sensitive and resistant clones. The addition of the 411-glycan made the sensitive cl.12 virus partially or fully resistant to 7 of the 12 sera but had less effect on the other 5. The neutralization titers of these five were nevertheless substantially reduced by the mutation (S4 Fig). However, removing the 411-glycan from the resistant cl.29 virus did not increase its neutralization sensitivity (Fig 7C). We conclude that the autologous NAbs induced by the CZA97 trimer target the V4 region and are often influenced by the absence or presence of the 411-glycan, but that other nearby, variable residues also affect the presentation of the epitope(s). Additional mutant viruses are required to delineate the epitope more fully.

### Heterologous neutralization assessed against a wider panel of viruses

To assess the breadth of neutralization, we tested sera from all 40 rabbits at weeks 22 and 26 and, for the appropriate groups also at weeks 50, 62 and 75, against a panel of 9 heterologous Tier-2 viruses (Table 2). Sera from 25 of the 40 rabbits detectably neutralized at least one test virus at one or more time points when an IC_50_ cutoff of > 20 was used, and 12 sera did so when a more rigorous cut-off of > 40 was used. There were four examples of heterologous NAb titers > 100 (of three different sera). Several of the heterologous responses were detected at the week-22 time point (2 weeks after the third immunization), but none at week 26 (2 weeks after the fourth). A few additional positive responses were seen at weeks 50 and 60, but none persisted until week 62; several more, but still sporadic, responses were recorded at weeks 73 and 75.

**Table 2:** Neutralization of heterologous Tier-2 viruses.

Virus		25710–2.43	TRO.11	BJOX002000.03.2	X1632-S2-B10	Ce1176_A3	246-F3_C10_2	CH119.10	Ce703010217_B6	CNE55
Clade/CRF		Clade C	Clade B	CRF07_BC	Clade B	Clade C	Clade AC	CRF07_BC	Clade A	CRF01_AE
Tier		Tier 2	Tier 2	Tier 2	Tier 2	Tier 2	Tier 2	Tier 2	Tier 2	Tier 2
Rabbit ID-group	Week									
5713–1	22	32 ^a^	39	- ^a^	-	-	-	-	-	-
	50	25	22	-	-	-	-	-	-	-
	60	-	-	-	25	23	33	24	20	**42**
5714–1	60	-	-	-	-	-	26	-	-	39
5715–1	50	-	-	-	-	-	-	27	-	-
	60	-	-	-	-	-	30	-	-	-
5716–1	60	-	-	-	-	-	33	24	-	26
	73	21	26	-	-	-	-	-	-	-
5724–3	22	37	32	-	-	30	28	24	-	-
5725–3	50	-		-	-	-	-	28	-	-
5726–3	22	**70**	**43**	-	-	-	-	-	-	-
	60	-	23	-	-	-	23	-	-	-
5727–3	60	-	32	-	-	-	28	-	-	-
	73	28	33	33	32	30	31	29	33	31
5728–4	22	**120**	**43**	-	23	-	-	-	-	-
	50	**46**	21	-	24	39	-	21	-	-
	60	23		-	-	-	29	23	-	-
5729–4	22	34	39	21	-	-	22	-	-	-
5732–4	22	**150**	**76**	-	22	-	-	-	-	-
	50	32	32	-	-	-	-	-	-	-
	60	**63**	34	-	-	27	34	33	-	29
5749–8	73	25	26	26	-	22	26	26	24	-
5750–8	22	**87**	**64**	-	-	-	-	-	-	-
	60	-		-	-	-	28	24	21	-
5751–8	60	38	**57**	-	21	**52**	**49**	**51**	**44**	**59**
5752–8	60	-	23	-	-	28	33	33	26	-
5718–2	73	22	-	-	-	-	24	31	27	-
5719–2	50	23	29	-	35	29	21	35	30	28
5721–2	60	-	26	-	-	-	-	-	-	-
	75	30	-	**49**	21	-	-	25	-	-
5733–5	50	33		-	-	**65**	-	-	-	-
	60	29		-	-	30	27	29	21	23
5734–5	50	-	22	-	-	25	26	27	-	-
	73	-	27	-	-	-	21	-	-	-
	75	-	25	-	-	-	23	-	-	-
5735–5	50	**154**	**83**	22	**48**	**233**	24	32	-	**68**
	73	21	26	21	**-**	**-**	24	-	22	**-**
5736–5	75	25	28	-	24	-	27	24	-	-
5738–6	22	-	35	-	-	-	20	-	-	-
5739–6	60	30	29	20	-	35	29	28	27	-
5741–6	22	**78**	**59**	-	-	22	-	-	-	-
	50	-	-	-	-	28	-	21	-	-

^*a*^ The lowest serum dilution tested in the neutralization assay was 1/20. A - symbol indicates that no neutralization was detectable at this serum dilution, i.e. a titer of < 20. Titers > 40 are highlighted in bold and are considered meaningful. Samples from weeks 22, 26, 50, 60, 62 and 75 were tested. The sera from the following 18 rabbits were negative against all 9 viruses at every time point tested: #5717–1, #5723–3, #5730–4, #5731–4, #5743–7, #5744–7, #5745–7, #5746–7, #5747–7, #5748–8, #5720–2, #5722–2, #573–5, #5740–6 and #5742–6.

The three most frequently neutralized viruses in the panel were: 25710–2.43 (clade C), TRO.11 (clade B), and Ce1176_A3 (clade C), while the least frequently hit was BJOX002000.03.2 (CRF07_BC). The glycan sites in the Env proteins of these isolates are shown in S1D Fig. We note that virus 25710–2.43 lacks the 241-glycan but has the sequence KVS at that site. However, two of the three sera that neutralized 25710–2.43 at a titer >40 failed to neutralize the BG505-S241K mutant (#5726–3 at week 22 and #5750–8 at week 22; Table 2 and S3A Table). The interpretation is that the heterologous neutralization is probably not specific for a glycan hole at the 241-site. Otherwise, there was no apparent pattern in the cross-neutralization data with respect to what trimers had been used to immunize the rabbits or the autologous NAb titers that were induced.

### Trimer-binding antibody responses

**Fig 9:**
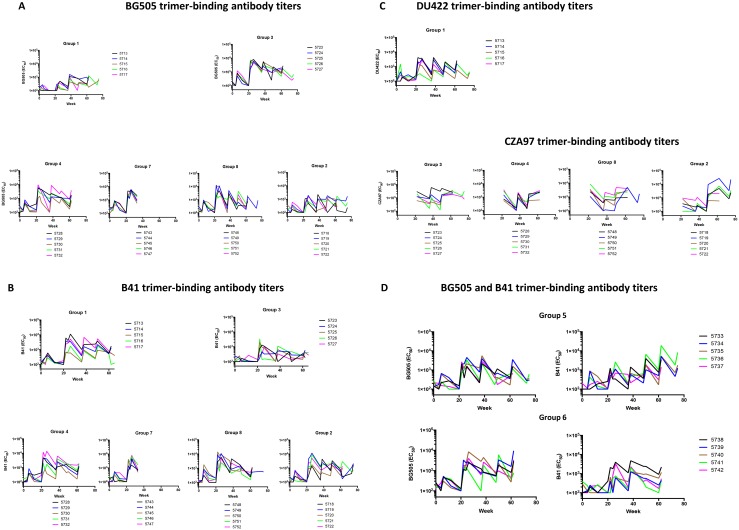
Trimer-binding antibody titers induced by trimer immunizations. The serum dilutions giving half-maximal binding (EC_50_) are plotted on the y-axes and the time of sampling on the x-axes for sera from individual rabbits (color-coded) in groups 1, 2, 3, 4, 7 and 8. The respective trimers used in the binding assays are **A**, BG505; **B**, B41; **C**, DU422 (group 1) and CZA97 (groups 2, 3, 4, 7 and 8). **D**, The corresponding plots for the sera from sequentially immunized rabbits (groups 5 and 6) show antibody binding to BG505 and B41 trimers.

We used ELISA to determine the half-maximal binding antibody titers (EC_50_) to all four of the trimer immunogens (Fig 9). In contrast to the NAb responses, autologous trimer-binding antibodies were detectable in sera from all rabbits. Cross-reactive binding antibodies, including cross-clade, were commonly observed. The decay of the trimer-binding antibody titers in the resting period between weeks 24 and 36 was more variable than the autologous NAb responses, although titer drops of ~10-fold were again frequently seen. The rates of decay could not be quantified in detail because of the paucity of time points, but were generally high.

**Fig 10:**
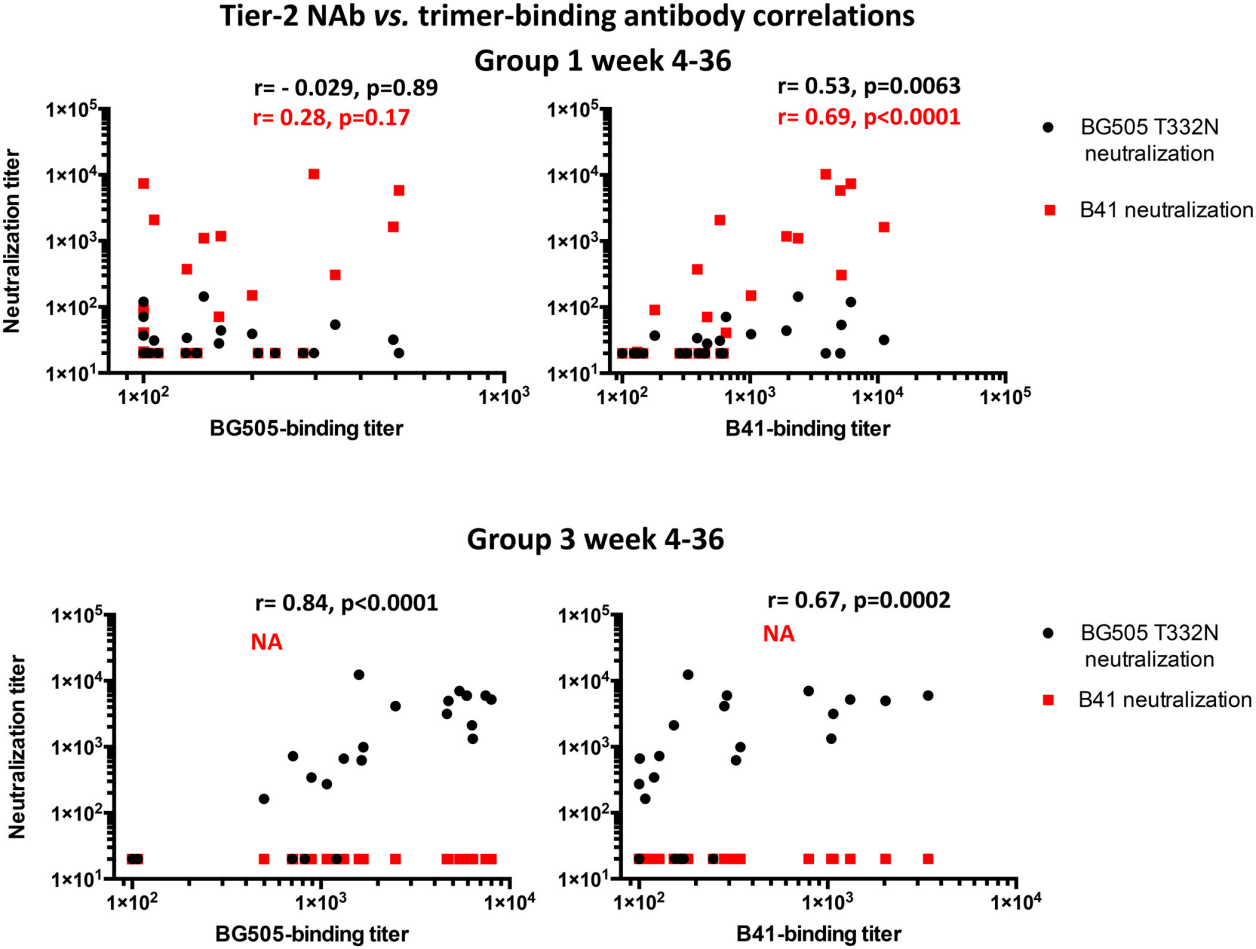
Correlations between trimer-binding and neutralizing antibody titers. The scatterplots show neutralization titers on the y-axes and the trimer-binding antibody titers on the x-axes. Within each plot the symbols corresponding to neutralization of the BG505.T332N and B41 viruses are color-coded as indicated on the figure panels. Spearman correlation coefficients (r-values) and the corresponding significances (p-values) are color-coded analogously. Correlation analyses were performed for both BG505 and B41 NAb and binding antibody titers for sera from the early monovalent immunogen regimens during weeks 4–36 (top panel, group 1; lower panel, group 3).

For simplicity, correlation analyses for the binding antibody and autologous NAb responses were restricted to the monovalent immunogen groups 1 and 3 (Fig 10). These rabbits received first either the BG505 or the B41 trimer, and later either the DU422 or CZA97 clade C trimer (Fig 1A). NAb responses during the period of only BG505 or B41 immunizations correlated better with the autologous than the heterologous trimer-binding antibody titers, although in group 3, a good correlation was observed between the BG505 autologous NAb titers and the B41 heterologous binding antibody titers (for correlation coefficients and significances, see Fig 10). There was a wide spectrum of heterologous trimer-binding antibody titers in sera that lacked any heterologous NAbs, ranging up to 500 for BG505 trimers in group 1 and up to 3400 for B41 trimers in group 3 (Fig 10). For correlations between binding antibody titers to trimers and NAbs for the clade A, B and C trimers and viruses from weeks 38–75, see S1 Text and S5 Fig.

Overall, we conclude that the various immunizing trimers induce substantial amounts of antibodies that have at least moderate affinities for autologous and heterologous trimers, but that lack the ability to neutralize genetically diverge Tier-2 viruses consistently. The extent to which V3 non-neutralizing antibodies contribute to the reactivity in the ELISA we used to measure trimer-binding antibodies will also need to be considered (5, 10). The strongest correlations between autologous binding and neutralizing antibody titers were seen after the BG505 and B41 monovalent trimer immunizations, and thus support the conclusions we previously drew about how the soluble SOSIP.664 immunogens are antigenic and structural mimics of virion-associated functional trimers [5, 49]. Furthermore, the correlations for DU422 trimer binding and the heterologous or cross-boosted NAb responses in group 1 suggest that the NAb specificities induced by the initial B41 trimer remained dominant throughout the period of heterologous (DU422) trimer boosting. At the same time, the NAb response broadened to cover a third virus (i.e., BG505.T332N) in animals that had not been immunized with that trimer. As there was no binding *vs*. neutralizing antibody correlation for DU422 itself, which was poorly neutralized, the data may indicate that the immune response to the boosting DU422 trimer was focused on previously seen epitopes. Additional studies would be needed to explore these various ideas.

## Discussion

Native-like soluble trimers, exemplified by SOSIP.664 and next-generation derivatives, are now being used to explore how bNAbs may eventually be induced by immunization [24, 26]. Studies of the immune responses to these trimers in various animals serve to identify which approaches are the more promising. In other words, how can the tool kit provided by the creation of native-like trimers be used most efficiently? This rabbit study was designed to generate information on the immunogenicity of multiple trimers, delivered sequentially or simultaneously. As noted above, the original design of some study groups was compromised by contamination that we detected while the initial set of immunizations was in progress. Other groups were unaffected, and we believe that there remains considerable value in the information derived from this study. One key point relating to the robustness of any conclusions is the limitation imposed by using group sizes of only 5 rabbits. With autologous NAb titers that vary in magnitude from undetectable to >10,000, random skews are inevitable. Moreover, the non-responsiveness of some rabbits, for reasons that are not understood, can also complicate data interpretations.

Rabbits respond to immunization with either BG505 or B41 trimers by inducing autologous NAbs against the corresponding Tier-2 viruses, as well as cross-reactive NAbs to Tier-1 viruses [5]. The latter responses, which are predominantly directed against V3, can be reduced by trimer-stabilization strategies that decrease the antigenicity and immunogenicity of V3 and other non-NAb epitopes [16]. However, to date the unmodified and stabilized versions of the BG505, B41 or other SOSIP.664 trimers have not induced substantial titers of bNAbs against heterologous Tier-2 viruses when delivered alone [5, 16, 51]. Can using more than one trimer improve matters in this regard?

When rabbits were immunized sequentially with the BG505 and B41 SOSIP.664 trimers they eventually generated autologous Tier-2 NAbs against both viruses. The consistency and high titers of these NAb responses were little different from when the same trimers were administered alone. Thus, rabbits can respond efficiently to more than one native-like trimer, when each is given three times over a prolonged period (~20 weeks). Fewer immunizations do not generate consistent and strong responses; we have not yet explored the minimum rest period required for the third immunization to be optimally effective. After the later clade C trimer boosts, several rabbits given three different trimers raised autologous NAbs to each one at various times. The response to the first trimer did not interfere markedly with the ability to raise autologous NAbs to the second one. In some cases, there was evidence for cross boosting, in that a second trimer modestly increased previously primed NAb titers to the first; this effect was observed most consistently when clade C trimers were given as heterologous boosts, particularly in respect to boosting the BG505.T332N NAb response. Both clade C trimers were immunogenic for autologous Tier-2 NAb responses, CZA97 being markedly more so than DU422. We have not yet obtained data on the *de novo* responses to these two trimers in rabbits, but it seems unlikely they would be very different from what was seen in the present experiment. The strong responses to the CZA97 SOSIP.664 trimers stand in marked contrast to the inability of uncleaved, non-native CZA97 gp140-Foldon proteins to induce autologous NAbs in immunized animals [1, 4, 52]. Taken together, the various observations on sequentially delivered trimers are relevant for devising how to use multiple trimer variants derived from a single genetic lineage; and perhaps also to immunization strategies involving priming with a germline bNAb-reactive trimer, followed by boosting with a more evolved trimer [24, 26, 53].

When BG505- and B41-based trimers were co-delivered at similar doses, autologous Tier-2 NAbs were raised against both viruses with no strong evidence for interference. Whether more substantial interference can arise in tri- and tetravalent immunizations will be determined from ongoing studies. The available evidence does not allow any strong conclusions about whether it is better to deliver two trimers each at the normal dose or at half of it (e.g., at 2 x 15 μg or 2 x 30 μg if the normal dose is 30 μg). The best way to co-deliver multiple (3 or more) trimers as a cocktail is being assessed experimentally.

We saw no sign, however, that sequential or simultaneous immunizations with two or three trimers was sufficient to generate consistent, high-level heterologous neutralization breadth at the Tier-2 level. Developing a bNAb response is unlikely to be as simple as mixing a cocktail, which is not to say that using more than one flavor of trimer has no value. When boosting previously primed responses, for example in germline antibody-targeting strategies, that approach may be exactly what is required. Nevertheless, we did detect NAbs against the BG505.T332N virus at substantial titers in several rabbits immunized with clade B and then clade C trimers. Thus, the NAbs against this Tier-2 virus arose in animals that had never seen BG505 SOSIP.664 trimers, and in some cases these NAbs appeared to target the 241/289 glycan hole of the BG505.T332N virus. However, the overall breadth of the Tier-2 response was limited to sporadic neutralization of heterologous viruses at titers that were generally <100.

We mapped autologous NAb responses to the BG505, B41 and CZA97 trimers to holes in their glycan shields, which supports an earlier suggestion about the nature of neutralization vulnerabilities for Tier-2 viruses [50]. Our new findings clearly supersede our earlier efforts to map the autologous NAb responses to BG505 trimers [5]. We can now show that the responses to these trimers in at least 17 of 30 rabbits target a glycan hole that is blocked by addition of a glycan at positions 241 and/or 289. Modeling shows that these two sites are closely located on the BG505 SOSIP.664 trimer structure and implies that each inserted glycan may partially occlude the same hole, from different directions (Fig 8). The data on the resistant phenotype of the BG505-S241K mutant, reinforced by the comparison of the MG505 cl.A2 and cl.A2-K241S viruses, indicate that serine-241 makes a major contribution to the autologous NAb epitope seen in these 17 rabbits. Whether additional NAb epitopes contribute to the overall response in the other 13 animals is not yet known, but any such site must be affected directly or indirectly by the additional sequence differences present in the MG505 clones (resistant) compared with BG505.T332N (sensitive). Here, variation in the C3 region between residues 354 and 363 may play a key role, perhaps by affecting how the 241/289 region of the trimer is presented [5]. Antibodies directed to gp120-gp41 interface epitopes and that directly or indirectly interfere with CD4 binding have been implicated in the autologous NAb response to BG505 trimers in the guinea pig [50]. How this antibody specificity relates to those described here remains to be determined.

We note that the lack of the 241-glycan creates a site of vulnerability on the BG505 virus and a corresponding immunogenic site on the BG505 trimer, and that no such exposed site is present on the maternal MG505 virus. It seems plausible that changes at position-241, perhaps in concert with others arising elsewhere (e.g., in C3), are involved in neutralization escape in this particular virus lineage. Because most circulating HIV-1 strains (~97%) resemble MG505 by bearing a glycan at the position-241 site they will be resistant to the induced antibodies, which is what we observed when the rabbit sera were tested against a Tier-2 virus panel. Moreover, even if a glycan hole were present at position-241 on a particular virus, the identity of the now-exposed residues at the base of the hole would also be important. Thus, the BG505.T332N S241K mutant, which lacks the glycan but contains a point substitution at position-241 compared with the trimer immunogen, was resistant to many rabbit sera. Once more, the sequence diversity of HIV-1 will be a formidable obstacle to vaccine development.

The rabbit response to the B41 trimers was predominantly directed against a glycan hole at residue-289 in C2 (where 60% of HIV-1 isolates have a glycan), while that to the CZA97 trimers seems centered on the V4 region and is likely to involve a glycan hole at position-411. Studies with additional mutant viruses would be required to define these epitopes in greater detail. We have not yet mapped the autologous NAb response induced in 2 of the 5 rabbits given the DU422 trimers. The finding that the BG505.T332N and B41 viruses share a glycan hole that is impeded by the introduction of a glycan at position-289 may be relevant to understanding some observations of heterologous boosting and heterologous cross-neutralization. Thus, in the group-1 animals, we observed that immunization with B41-based trimers followed by boosting with DU422 trimers induced NAbs that neutralized the BG505.T332N virus. These heterologous NAbs targeted the same 241-glycan hole as the autologous NAbs raised against the BG505 trimers. Perhaps some antibodies induced by the immunogenic 289-glycan hole on the B41 trimers can recognize the similar hole on the BG505.T332N virus, and hence drive a degree of cross-neutralization. Cross boosting of the BG505.T332N and B41 NAb responses by the clade C trimers, again in a manner sensitive to the 289-glycan in both cases, may be rooted in the same mechanism. Among the three most frequently, but still sporadically, neutralized heterologous Tier-2 viruses we note that 25710–2.43 lacks the 241-glycan. Various observations in individual rabbits or sub-groups are not easily understood, including some of the boosting effects of the DU422 trimers that have glycans at both positions 241 and 289. Overall, these various ideas are tentative and would require additional experiments to confirm or refute.

Although our overall emphasis is on suppressing rather than inducing Tier-1 NAb responses, we did quantify them [16]. The kinetics of the Tier-1 NAb responses induced by the BG505 and B41/B41-D7324 trimers clearly show that these antibodies are detectable earlier than, and not correlated with, the Tier-2 autologous NAb responses in the same animals (Figs 2 and 8 and S2 Fig). This finding is generally consistent with our previous conclusions (5). The lack of correlation between the Tier-1 and autologous Tier-2 NAb responses, both temporally within a rabbit and among a group of rabbits, should now be considered in light of what we are learning about the epitopes involved. Thus, Tier-1 NAb responses are dominated by antibodies to V3, a region of Env that contains a cluster of continuous, peptide epitopes that are relatively devoid of any shielding glycans. In contrast, we now show that the autologous Tier-2 NAb epitopes involve holes in the glycan shield that are presumably influenced by the surrounding glycans. We suggest that antibody responses to these different categories of neutralization epitopes may not be elicited or boosted in the same way. If so, what has been learned from earlier generations of Env glycoproteins that induce predominantly V3-NAbs would not be informative about how NAb responses to Tier-2 epitopes are best elicited; different immunization regimens and/or adjuvants may be needed.

The trimer genotype is now emerging as an additional important variable, in this and in other experiments that we have been conducting. For example, we observed here that, in the heterologous boost context, the CZA97 trimer was more immunogenic than its DU422 counterpart. Moreover, a few rabbits were non-responders to CZA97, DU422 and B41 trimers but none to BG505, through influences that we are yet to understand. We are now generating and testing hypotheses about what specific features of individual trimers, and the corresponding viruses, most influence the induction of autologous Tier-2 NAb responses. Whether the same characteristics will also affect how different trimers cross boost existing NAb responses, or induce heterologous responses, will also need to be understood. Host factors are also important. Within a group of rabbits, there can be a wide range (2–3 orders of magnitude) of autologous NAb titers to the same trimer, and some animals do not respond at all. Whether an animal responds or not to a particular trimer immunogen by generating autologous Tier-2 NAbs, and the magnitude of any response, may be influenced by the genetics of both the antibody repertoire and of factors that affect the development of humoral immunity more generally. Whether different genetic factors affect the generation of Tier-1 and autologous Tier-2 NAb responses should also be considered in light of the different characteristics of the relevant epitopes. An influence of host genetic factors will not, of course, be unique to rabbits, although the identities of the factors involved may be species-dependent.

This series of experiments was performed in rabbits, because the strong and consistent autologous NAb responses to the BG505 and B41 trimers in this species allow endpoints to be determined [5]. In contrast, BG505 trimers are only moderately immunogenic in macaques [5] and do not induce any autologous NAb response in mice that can be quantified [54]. Guinea pigs, however, do respond to BG505 trimers about as well as rabbits [51]. Which species provide the best models for predicting human responses to these trimers will be better understood as key immunological parameters become identified, and if and when data from human studies allow a retrospective comparison. We are now using the techniques reported here to map the targets of the autologous NAbs induced by BG505 SOSIP trimers in responding macaques and guinea pigs and determine whether they are different from those we find in the rabbit. Based on the current assumption that the macaque is the most likely of these species to mimic the human response, these ongoing studies may be of considerable value for identifying which, if any, small animal model is suitable for further testing of trimer immunogens.

In conclusion, the observations and inferences from this complex set of experiments may be a valuable guide on how best to design and use trimer-based immunogens in the quest to induce bNAb responses in humans. The limitations of the study, the restricted statistical power of small groups, and perhaps other issues inherent to the choice of model species, must be recognized. Nevertheless, the present results suggest that the induction of sufficient neutralization breadth will probably not be achieved through current regimens simply by combining a few native-like trimers into a cocktail without further advances in immunogen delivery methods. The combination approach may, however, be useful for boosting responses primed by germline bNAb-targeting trimers, particularly if our understanding of genotype-dependent influences on immunogenicity advances. Knowledge of the nature of the epitopes responsible for neutralization of Tier-2 viruses is likely to aid the design of immunogens intended to convert narrow-specificity NAb responses that, perhaps, evade glycans into broader ones that, in some cases, accommodate their presence. Thus, glycan holes may be the initial targets for several bNAbs in their ontogeny in the infected human [55–57] and the continued removal and insertion of glycans at particular positions during natural infection may eventually lead to the emergence of broader responses. If so, better understanding the characteristics of epitopes and immunogenic sites, both natural and engineered variants, would be useful. Among other strategies to consider are the targeted opening or closing of glycan holes to try to alter the immunogenicity of trimers beneficially, as well as more sophisticated, structure-guided approaches towards accommodating glycans that abut key epitopes.

## Supporting Information

S1 Text Supporting Information Text. (DOC)

S1 Fig Sequence alignments for key viruses and clones used in this study. Amino-acid differences are highlighted in gray and variable Env regions in yellow. Glycan sites are indicated in red, while the absence of a glycan (a glycan hole) in relation to comparator sequence or other isolates is highlighted in green. In panel **D,** the glycan holes are defined in relation to the majority of the Env sequences in the Los Alamos Sequence Data Base (http://www.hiv.lanl.gov).
(PDF)

S2 Fig Lack of correlation between Tier-1 and autologous Tier-2 NAb responses. Top panels: NAb titers at week-22 are compared for the Tier-1 viruses MN.3 (left) and MW965.26 (right) and the autologous Tier-2 viruses BG505.T332N (excluding group 1 because of weak neutralizing responses) and B41 (excluding groups 3 and 6, which were negative for neutralization), as indicated. Bottom panels: NAb titers at week-62 are compared for the Tier-1 viruses MN.3 (left) and MW965.26 (right) and the autologous Tier-2 viruses BG505.T332N, B41, DU422 (group 1) and CZA97 (groups 2, 3, 4 and 8). The week-62 BG505.T3322N and B41 correlations with Tier-1 titers involve all the groups of rabbits bled at that time point, since some cross-neutralizing responses had arisen against BG505.T332N, albeit generally at low titers. No cross-neutralization of B41 was observed at week-62, however. Spearman-correlation analyses of these comparisons are recorded in S2 Table.
(TIFF)

S3 Fig Single *vs*. dual simultaneous immunizations with clade A and B trimers. NAb titers against the BG505.T332N (left) and B41 (right) viruses are compared at the peak (week-22) of the responses to single or dual immunizations as follows: group 1 (30 μg clade B trimer), group 3 (30 μg clade A trimer), group 4 (30 μg total, 60% clade A and 40% clade B), group 7 (30 μg total, 40% clade A and 60% clade B), group 8 (90 μg total, 40% clade A and 60% clade B) and group-2 (30 μg total, 20% clade A and 80% clade B). The NAb titers (IC_50_, mean ± s.e.m. on a log-scale) are shown on the y-axis for the groups of five rabbits indicated on the category axis.
(TIFF)

S4 Fig Neutralization titer differences among WT and mutant viruses, or clonal variants, from clades A and C. The values shown represent the reductions in neutralization titer for (A) BG505.T332N virus mutants relative to wild-type (WT, = 100%); or (B) CZA97 clones or mutants thereof relative to cl.12 (= 100%). The sera listed are the subset from the groups presented in Fig 7 for which titration curves revealed significant reductions in neutralization sensitivity of various mutants or clones that were not apparent at a single serum dilution of 1/50 (BG505.T332N) or 1/60 (CZA97). The numbers in brackets in orange cells represent relative titers of sera that neutralized the respective viruses to an extent of <25% of WT (Fig 7); in those cases the titration was not necessary to show a neutralization difference.
(TIFF)

S5 Fig Relationships between trimer-binding antibody and NAb titers. The scatterplots show NAb titers on the y-axes and the antibody binding titers to SOSIP.664-D7324 trimers on the x-axes. Within each plot the symbols corresponding to neutralization of the BG505.T332N, B41, DU422 and CZA97 viruses are color-coded as indicated on the figure panels. Spearman correlation coefficients (r-values) and the corresponding significances (p-values) are color-coded analogously. The scatter plots show the BG505, B41 and DU422 or CZA97 NAb and binding antibody titers for groups-1 and -3, during the period of DU422 or CZA97 trimer boosting immunizations from weeks 38–62 (top panel, group-1, DU422 boosting; lower panel, group-3, CZA97 boosting).
(PPTX)

S1 Table Antibody responses to the D7324-epitope tag on the B41-D7324 trimer. (DOC)

S2 Table Correlation coefficients for comparisons between Tier-1 and autologous Tier-2 NAb titers. (DOC)

S3 Table Mapping of autologous Tier-2 NAb responses. (DOC)
