# Supplementary material for: Sequential and Simultaneous Immunization of Rabbits with HIV-1 Envelope Glycoprotein SOSIP.664 Trimers from Clades A, B and C
Source: PLoS Pathog. 2016 Sep 14;12(9):e1005864. doi: 10.1371/journal.ppat.1005864 (PMC5023125; doi:10.1371/journal.ppat.1005864)
Supplement: S1 Text — (DOC) [file ppat.1005864.s001.doc]

**Supplementary Information**

**Supplementary Text**

*The presence of the D7324-epitope tag does not markedly influence trimer immunogenicity*

For group 1, the immunogen was the B41 trimer (30 µg), and for group 2, it was a mixture of the B41-D7324 trimer (~24 µg) with some contaminant BG505 trimer (~6 µg). The B41 NAb responses did not differ significantly between group-1 and group-2 rabbits (Figure 2B, peak titers, week 22, p=0.69). Thus, neither the presence of the BG505 trimer (~20% of the total) in group-2 nor the C-terminal D7324-tag on the B41-D7324 trimer significantly influenced the development of the autologous B41 NAb response. Observations made in mouse immunogenicity studies indicate that non-NAbs to the exposed base of BG505 SOSIP.664 trimers may have an adverse influence on the overall response in that species (1). As the D7324-epitope tag is located at the base of the B41-D7324 trimers, we assessed whether binding Abs were raised against this tag in rabbits. We did so by coating wells with a peptide corresponding to the D7324-tag and adding week-26 sera from 16 different rabbits at dilutions from 20- to 50,000-fold. No significant binding was observed with any of the sera, compared with the background signal for a sequence-scrambled control peptide, regardless of whether the rabbits had been immunized with trimers bearing (#5718-5722, 5728, 5729, 5743, 5744) or lacking (#5713-5717, 5723, 5724) the D7324-tag. In parallel control assays, the D7324 antibody bound strongly to the coated peptide (EC_50_, 10 ng/ml), as did an IgG pool of sera from HIV-1-infected humans (EC_50_, 84 µg/ml) (Table S1). The human serum antibody reactivity reflects the immunogenicity of the C5-region of gp120, from which the D7324-sequence is derived.

Thus, we could find no evidence that the D7324-tag on B41-D7324 trimers is immunogenic in rabbits, which contrasts with the strong antibody response elicited by larger C-terminal additions to Env proteins, such as Foldon (2). Overall, the use of more than one trimer immunogen in groups-2, -4 and -7, the small group sizes of five rabbits, and the lack of any response to the D7324-tag preclude us from drawing further conclusions on whether non-NAbs elicited to the trimer base can affect the quality and extent of autologous NAbs in rabbits.

*Single vs. dual simultaneous immunizations with clade A and B trimers (groups 1 and 3 compared with groups 4, 7, 8 and 2)*

We investigated whether two genetically divergent trimers (i.e., from two different clades, A and B) were both immunogenic when co-delivered, and how dosing affects the response. Thus, we compared the autologous NAb responses to single trimers described above for groups 1 (30 µg clade B trimer) and 3 (30 µg clade A trimer) with responses to mixtures of clade A (BG505) and clade B (B41) trimers. The mixtures were delivered at two different total doses of 30 µg (groups 4, 7 and 2) and 90 µg (group 8), at weeks 0, 4, 20 and 24 (Fig. 1B). The 30-µg total dose was also used for single trimer-immunizations. The immunogen composition in groups 7 (30 µg total) and 8 (90 µg total) was 40% clade A and 60% clade B, in group 4 (30 µg total) it was 60% clade A and 40% clade B, and in group 2 it was 20% clade A and 80% clade B (30 µg total) (see the schematic representation in Figure 1).

The autologous NAb responses in these groups are shown in S3 Figure. At the peak of the response (week-22), the groups differed in BG505.T332N NAb titers (Kruskal-Wallis’s test, p=0.0045), but only in that group-3 had higher titers than group-2 (post-test, p<0.01). Thus, the trimer dose used to immunize group-7 (low dose; 30 µg total) *vs.* group-8 (high dose; 90 µg total) rabbits, at identical clade A to B trimer ratios of 40/60, did not detectably affect the resulting BG505.T332N autologous NAb titers. Furthermore, since the responses in groups-3 and -8 were similar, the simultaneous addition of a somewhat larger dose of the clade B trimer did not compromise the response to the clade A trimer given at approximately the standard dose. The data nevertheless suggest that the autologous BG505.T332N NAb response to the lowest dose of the clade A trimer was suppressed when it was simultaneously delivered with a higher dose of the clade B trimer (group 2); even, then, however, autologous BG505.T332N NAbs were readily detectable in 4 of 5 rabbits. The BG505.T332N NAb titer reduction might be due to a limited interference by the clade B trimer, but it is also possible that the dose of the clade A trimer in this particular mixture (6 µg) was below optimum for inducing autologous NAb responses. In support of the former explanation, the BG505.T332N NAb titers for group 4 tended to be weaker than for group 3; while this trend was not statistically significant at week-22, it was modestly significant at week-26 (p<0.05). In contrast to that trend, however, the corresponding NAb response for group-7 was higher than for group-4, despite the fact that group-7 received a higher ratio of clade B to clade A trimers (at the same total dose). More focused studies will be needed to determine whether any of these various and generally modest BG505.T332N NAb titer differences are attributable to genuine interference effects between co-delivered trimers, to dose-effects, or to random variations associated with group sizes of 5 animals.

In contrast to the above variations in the BG505.T332N NAb responses among the rabbit groups, comparison of the B41 NAb titers in the same groups at the same week-22 and week-26 time points showed no differences among them (Kruskal-Wallis’s test, p=0.62 and p=0.68) (S3 Figure). Hence no suppressive effects of the clade A trimer on the autologous B41 NAb response could be discerned.

Overall, this part of the study suggests that the total trimer immunogen dose of 30 µg is sufficient to induce autologous NAbs in rabbits. At this total dose, two different trimers can be co-delivered in approximately equimolar amounts without substantial interference between them. In summary, a bivalent mixture of trimers usually elicited autologous NAb responses to both Tier-2 viruses.

*Variation in autologous NAb specificities over time*

We also studied how the specificity of the BG505.T332N NAb response changed over time in some rabbits. For example, neutralization of the various 241- and 289-glycan knock-in viruses increased over time for rabbits #5713-1, #5715-1, #5733-5, #5734-5, #5735-5 and #5739-6, but decreased for #5727-3, #5748-8 and #5751-8 (Table S3). This pattern of responses was not obviously linked to boosting with clade C trimers that contain glycans at positions 241 and 289; increased sensitivity over time occurred in rabbits from groups-5 and -6 that did not receive clade C trimers and, conversely, decreased sensitivity was seen in rabbits from groups 3 and 8 that did receive such a boost.

A comparison of all the groups of rabbits summarized in Figure 7A suggested that glycan knock-in effects among them were not random (Kruskal-Wallis, p=0.058). Specifically, the peak-titer sera from group-6 neutralized the 241+289 double glycan knock-in mutant more effectively than the group-5 sera (post test to the Kruskal-Wallis, p<0.05). This observation suggests that the dominant autologous NAb specificity differs between the two groups. Over time, titers increased in both groups, but the 241- and/or 289-glycan knock-in mutants were only completely neutralized by the group-6 sera (Table S3). We note that the group-6 rabbits received three B41-trimer-containing boosts during weeks 24-36, whereas group-5 received three BG505-trimer boosts over the same time. It is also apparent that the BG505.T332N NAb titers for group-6 increased after week 38, but they fell over the same time for group-5 (Fig. 5A and C). It is possible that continual boosting of the BG505.T332N NAb responses with trimers that carry the 241 glycan (e.g., B41) may ultimately overcome the impediments posed by these glycans. However, additional studies are needed to explore the mechanisms underlying these various observations, and hence to improve our knowledge of how to generate and boost Tier-2 NAb responses more generally.

*Correlations between antibody binding to Env trimers and Tier-2 NAb responses*

Correlations between antibody binding to SOSIP.664-D7324 trimers and NAb titers in the period of DU422 trimer boosting of group-1 rabbits, i.e. from weeks 38-75, were also assessed. There was no correlation between the DU422 autologous NAb and antibody binding titers for the two NAb-responsive rabbits (#5715, #5716), and the same was true for the other trimer-binding antibody titers. However, the heterologous BG505.T332N and the cross-boosted B41 NAb responses at the same time points did correlate weakly with the DU422 binding antibody titers (S5 Figure). The DU422 cross-boosted, autologous B41 NAb response was unique in its tendency to correlate with the B41-trimer binding antibody titers. Thus, for the same sera, the heterologous BG505 NAb response, which was primed by B41 trimers and boosted by DU422 trimers, correlated well only with DU422-trimer binding antibodies (S5 Figure). We did note that, unexpectedly, the autologous DU422 NAb titers correlated negatively with the B41-trimer-binding antibody titers.

In group-3, there were no positive correlations between the autologous trimer-binding antibody and NAb responses to the clade C CZA97 trimer from weeks 38 to 75 (S5 Figure). During that period, the BG505.T332N NAb titers for group-3 now failed to correlate with any measure of trimer binding (S5 Figure), although they correlated strongly with both BG505- and B41-trimer binding titers at earlier times (Fig. 10).

**Supplementary Figures**

**S1 Figure. Sequence alignments for key viruses and clones used in this study**

Amino-acid differences are highlighted in gray and variable Env regions in yellow. Glycan sites are indicated in red, while the absence of a glycan (a glycan hole) in relation to comparator sequence or other isolates is highlighted in green. In panel **D,** the glycan holes are defined in relation to the majority of the Env sequences in the Los Alamos Sequence Data Base (http://www.hiv.lanl.gov).

**S2 Figure . Lack of correlation between Tier-1 and autologous Tier-2 NAb responses.**

Top panels: NAb titers at week-22 are compared for the Tier-1 viruses MN.3 (left) and MW965.26 (right) and the autologous Tier-2 viruses BG505.T332N (excluding group 1 because of lack of neutralizing responses) and B41 (excluding groups 3 and 6, which were negative for neutralization), as indicated. Bottom panels: NAb titers at week-62 are compared for the Tier-1 viruses MN.3 (left) and MW965.26 (right) and the autologous Tier-2 viruses BG505.T332N, B41, DU422 (group 1) and CZA97 (groups 2, 3, 4 and 8). The week-62 BG505.T3322N and B41 correlations with Tier-1 titers involve all the groups of rabbits bled at that time point, since some cross-neutralizing responses had arisen against BG505.T332N, albeit generally at low titers. No cross-neutralization of B41 was observed at week-62, however. Spearman-correlation analyses of these comparisons are recorded in Table S2.

**S3 Figure. Single *vs.* dual simultaneous immunizations with clade A and B trimers.** NAb titers against the BG505.T332N (left) and B41 (right) viruses are compared at the peak (week-22) of the responses to single or dual immunizations as follows: group-1 (30 µg clade B trimer), group-3 (30 µg clade A trimer), group-4 (30 µg total, 60% clade A and 40% clade B), group-7 (30 µg total, 40% clade A and 60% clade B), group-8 (90 µg total, 40% clade A and 60% clade B) and group-2 (30 µg total, 20% clade A and 80% clade B). The NAb titers (IC_50_, mean ± s.e.m. on a log-scale) are shown on the y-axis for the groups of five rabbits indicated on the category axis.

**S4 Figure. Neutralization titer differences among WT and mutant viruses, or clonal variants, from clades A and C.** The values shown represent the reductions in neutralization titer for  (A) BG505.T332N virus mutants relative to wild-type (WT, =100%); or (B) CZA97 clones  or mutants thereof relative to cl.12 (=100%). The sera listed are the subset from the groups presented in Figure 7 for which titration curves revealed significant reductions in neutralization sensitivity of various mutants or clones that were not apparent at a single serum dilution of 1/50 (BG505.T332N) or 1/60 (CZA97).  The numbers in brackets in orange cells represent relative titers of sera that neutralized the respective viruses to an extent of <25% of WT (Figure 7); in those cases the titration was not necessary to show a neutralization difference.

**S5 Figure. Relationships between trimer-binding antibody and NAb titers.** The scatterplots show NAb titers on the y-axes and the antibody binding titers to SOSIP.664-D7324 trimers on the x-axes. Within each plot the symbols corresponding to neutralization of the BG505.T332N, B41, DU422 and CZA97 viruses are color-coded as indicated on the figure panels. Spearman correlation coefficients (r-values) and the corresponding significances (p-values) are color-coded analogously. The scatter plot plots show the BG505, B41 and DU422 or CZA97 NAb and binding antibody titers for groups-1 and -3, during the period of DU422 or CZA97 trimer boosting immunizations from weeks 38-62 (top panel, group-1, DU422 boosting; lower panel, group-3, CZA97 boosting).

**Supplementary References**

1. **Hu JK, Crampton JC, Cupo A, Ketas T, van Gils MJ, Sliepen K, de Taeye SW, Sok D, Ozorowski G, Deresa I, Stanfield R, Ward AB, Burton DR, Klasse PJ, Sanders RW, Moore JP, Crotty S.** 2015. Murine Antibody Responses to Cleaved Soluble HIV-1 Envelope Trimers Are Highly Restricted in Specificity. J Virol **89:**10383-10398.

2. **Sliepen K, van Montfort T, Melchers M, Isik G, Sanders RW.** 2015. Immunosilencing a highly immunogenic protein trimerization domain. J Biol Chem **290:**7436-7442.
