# Supplementary material for: Sequential and Simultaneous Immunization of Rabbits with HIV-1 Envelope Glycoprotein SOSIP.664 Trimers from Clades A, B and C
Source: PLoS Pathog. 2016 Sep 14;12(9):e1005864. doi: 10.1371/journal.ppat.1005864 (PMC5023125; doi:10.1371/journal.ppat.1005864)
Supplement: S1 Fig — Amino-acid differences are highlighted in gray and variable Env regions in yellow. Glycan sites are indicated in red, while the absence of a glycan (a glycan hole) in relation to comparator sequence or other isolates is highlighted in green. In panel D, the glycan holes are defined in relation to the majority of the Env sequences in the Los Alamos Sequence Data Base (http://www.hiv.lanl.gov). (PDF) [file ppat.1005864.s002.pdf]

S1 Figure. Sequence alignments for key viruses and clones used in this study

A. BG505.T332N *vs.* B41

```
BG505.T332N     MRVMGIQRNCQHLFRWGTMILGMIIICSAAENLWVTVYYGVPVWKDAETTLFCASDAKAY
B41             MRVMEIRRNCQHLWRGGILLLGILMICSAAK-KWVTVYYGVPVWKEATTTLFCASDAKAY

                                          88
BG505.T332N     ETEKHNVWATHACVPTDPNPQEIHLENVTEEFNMWKNNMVEQMHTDIISLWDQSLKPCVK
B41             DTEVHNVWATHACVPTDPNPQEIVLGNVTENFNMWKNNMVEQMHEDIISLWDQSLKPCVK

                     130 133 137         156 160
BG505.T332N     LTPLCVTLQCTNVTNNITDDMR-------------GELKNCSFNMTTELRDKKQKVYSLFY
B41             LTPLCVTLNCNNVNTNNTNNSTNATISDWEKMETGEMKNCSFNVTTSIRDKIKKEYALFY

                       190 190c  197
BG505.T332N     RLDVVQINENQGNRSNNSNKEYRLINCNTSAITQACPKVSFEPIPIHYCAPAGFAILKCK
B41             KLDVVPLE-NKNNINNTNITNYRLINCNTSVITQACPKVSFEPIPIHYCAPAGFAILKCN

                230 234 241          262      276      289
BG505.T332N     DKKFNGTGPCPSVSTVQCTHGIKPVVSTQLLLNGSLAEEEVMIRSENITNNAKNILVQFN
B41             SKTFNGSGPCTNVSTVQCTHGIRPVVSTQLLLNGSLAEEEIVIRSENITDNAKTIIVQLN

                   295 301                 332  339
BG505.T332N     TPVQINCTRPNNNTRKSIRIGPGQAFYATGDIIGDIRQAHCNVSKATWNETLGKVVKQLR
B41             EAVEINCTRPNNNTRKSIHIGPGRAFYATGDIIGNIRQAHCNISKARWNETLGQIVAKLE

                  355  363             386 392 398 406 411
BG505.T332N     KHFGNNTIIRFANSSGGDLEVTTHSFNCGGEFFYCNTSGLFNSTWISNTSVQGSNSTGSN
B41             EQFPNKTII-FNHSSGGDPEIVTHSFNCGGEFFYCNTTPLFNSTW--NNTRTDDYPTGGE

                                       448       462
BG505.T332N     DSITLPCRIKQIINMWQRIGQAMYAPPIQGVIRCVSNITGLILTRDGGSTNSTTETFRPG
B41             QNITLQCRIKQIINMWQGVGKAMYAPPIRGQIRCSSNITGLLLTRDGGRDQNGTETFRPG

                                           Vcleavage
BG505.T332N     GGDMRDNWRSELYKYKVVKIEPLGVAPTRAKRRVVGREKRAVGIGAVFLGFLGAAGSTMG
B41             GGNMRDNWRSELYKYKVVKIEPLGIAPTAAKRRVVQREKRAVGLGAFILGFLGAAGSTMG

BG505.T332N     AASMTLTVQARNLLSGIVQQQSNLLRAIEAQQHLLKLTVWGIKQLQARVLAVERYLRDQQ
B41             AASMALTVQARLLLSGIVQQQNNLLRAIEAQQHMLQLTVWGIKQLQARVLAVERYLRDQQ

                            611  618 625     637
BG505.T332N     LLGIWGCSGKLICTTNVPWNSSWSNRNLSEIWDNMTWLQWDKEISNYTQIIYGLLEESQN
B41             LLGIWGCSGKIICTTNVPWNDSWSNKTINEIWDNMTWMQWEKEIDNYTQHIYTLLEVSQI
```

```
                              SOSIP.664 truncation
BG505.T332N      QQEKNEQDLLALDKWASLWNWFDISNWLWYIKIFIMIVGGLIGLRIVFAVLSVIHRVRQG
B41              QQEKNEQELLELDKWDSLWNWFSISNWLWYIKIFIMIVGGLIGLRIVFTVLSIISRVRQG


BG505.T332N      YSPLSFQTHTPNPRGLDRPERIEEEDGEQDRGRSTRLVSGFLALAWDDLRSLCLFCYHRL
B41              YSPLSFQTLLPVPRGPDRPEGIEEEGGERDRDRSGPPVNGFLAIFWVDLRNLFLFLYHRL


BG505.T332N      RDFILIAARIVELLGHSSLKGLRLGWEGLKYLWNLLAYWGRELKISAINLFDTIAIAVAE
B41              RDLLLIAARIVELLGRR-------GWGILKYWWNLLQYWSQELKNSAVSLLNATAIAVAE


BG505.T332N      WTDRVIEIGQRLCRAFLHIPRRIRQGLERALL
B41              GTDRVIEVVQRIVRGILHIPTRIRQGLERALL
```

Unknown
Formatted: Font:(Default) Courier, Bold, Font color: Light Blue

B. BG505.T332N *vs.* MG505 cl.A2 and cl.H3

```
BG505.T332N      MRVMGIQRNCQHLFRWGTMILGMIIICSAAENLWVTVYYGVPVWKDAETTLFCASDAKAY
MG505.A2         MRVVGIQRNCQHLFRWGTMILGMIIICSAAENLWVTVYYGVPVWKDAETTLFCASDAKAY
MG505.H3         MRVMGIQMNCQHLFRWGTMILGMIIICSAAENLWVTVYYGVPVWKDAETTLFCASDAKAY

                                           88
BG505.T332N      ETEKHNVWATHACVPTDPNPQEIHLENVTEEFNMWKNNMVEQMHTDIISLWDQSLKPCVK
MG505.A2         ETEKHNVWATHACVPTDPNPQEIHLENVTEEFNMWKNSMVEQMHTDIISLWDQSLKPCVK
MG505.H3         KTEKHNVWATHACVPTDPNPQEIHLENVTEEFNMWKNSMVEQMHTDIISLWDQSLEPCVK

                        130 133  137        156 160
BG505.T332N      LTPLCVTLQCTN--VTNNITDDMRGELKNCSFNMTTELRDKKQKVYSLFYRLDVVQINEN
MG505.A2         LTPLCVTLQCTN--VTNNITDDMRGELKNCSFNMTTELRDKKQKVYSLFYRLDVIQINEN
MG505.H3         LTPLCVTLQCTNNVTNANITDDMRGELKNCSFNMTTELRDKKQKVYSLFYRLDVVQINEN

                  190 190c       197                                230 234
BG505.T332N      QGNRSNNSNKEYRLINCNTSAITQACPKVSFEPIPIHYCAPAGFAILKCKDKKFNGTGPC
MG505.A2         QGNGSNNSNKEYRLINCNTSAITQACPKVSFEPIPIHYCAPAGFAILKCKDKKFNGTGPC
MG505.H3         QGNSSKSSNKEYRLINCNTSAITQACPKVSFEPIPIHYCAPAGFAILKCKDKKFTGTGPC

                 241                  262           276          289    295
BG505.T332N      PSVSTVQCTHGIKPVVSTQLLLNGSLAEEEVMIRSENITNNAKNILVQFNTPVQINCTRP
MG505.A2         PKVSTVQCTHGIKPVVSTQLLLNGSLAEEEVMIRSENITNNAKNILVQFNTPVQINCTRP
MG505.H3         SNVSTVQCTHGIKPVVSTQLLLNGSLAEEEVMIRSENITNNAKNILVQFNTPVQINCTRP

                 301                           332    339             355
BG505.T332N      NNNTRKSIRIGPGQAFYATGDIIGDIRQAHCNVSKATWNETLGKVVKQLRKHFGNNTIIR
MG505.A2         NNNTRKSIRIGPGQAFYATGDIIGDIRQAHCTVSKATWNETLEKVVKQLRKHFGNNKTII
MG505.H3         NNNTRKSIHIGPGQAFYATGDIIGNIRQAQCTVSKATWNETLEKVVKQLREHFGNNTTII

                  363                   386   392   398    406    411
BG505.T332N      FANSSGGDLEVTTHSFNCGGEFFYCNTSGLFNSTWISNTSVQGSNSTGSNDSITLPCRIK
MG505.A2         FANSSGGDLEVTTHSFNCGGEFFYCNTSGLFNSTWNSNTSVQGSNSTGSNDSITLPCRIK
MG505.H3         FASSSGGDLEVTTHSFNCGGEFFYCNTSGLFNSTWISNTSVQGSNSTESSDTITLPCRIK
```

```
                                                448           462
BG505.T332N     QIINMWQRIGQAMYAPPIQGVIRCVSNITGLILTRDGGSTNSTTETFRPGGGDMRDNWRS
MG505.A2        QIINMWQRIGQAMYAPPIQGVIGCVSNITGLILTRDGGSTNSTTETFRPGGGDMRDNWRS
MG505.H3        QIINMWQRIGQAMYAPPIQGVIRCVSNITGLILTRDGGSNNSTNETFRPGGGDMRDNWRS
```

```
                                            Furin cleavage
BG505.T332N     ELYKYKVVKIEPLGVAPTRAKRRVVGREKRAVGIGAVFLGFLGAAGSTMGAASMTLTVQA
MG505.A2        ELYKYKVVKIEPLGVAPTRAKRRVVGREKRAVGIGAAFLGFLGAAGSTMGAASMTLTVQA
MG505.H3        ELYKYKVVKIEPLGVAPTRAKRRVVGREKRAVGIGAVFLGFLGAAGSTMGAASMTLTVQA
```

Unknown
Formatted: Font:(Default) Courier, Bold, Font color: Light Blue

```
BG505.T332N     RNLLSGIVQQQSNLLRAIEAQQHLLKLTVWGIKQLQARVLAVERYLRDQQLLGIWGCSGK
MG505.A2        RNLLSGIVQQQSNLLRAIEAQQHLLKLTVWGIKQLQARVLAVERYLRDQQLLGIWGCSGK
MG505.H3        RNLLSGIVQQQSNLLRAIEAQQHLLKLTVWGIKQLQARVLAVERYLRDQQLLGIWGCSGK
```

```
                        611    618    625          637
BG505.T332N     LICTTNVPWNSSWSNRNLSEIWDNMTWLQWDKEISNYTQIIYGLLEESQNQQEKNEQDLL
MG505.A2        LICTTNVPWNSSWSNKNLSEIWDNMTWLQWDKEISNYTQIIYGLLEESQNQQEKNEQDLL
MG505.H3        LICTTNVPWNSSWSNRNLSEIWDNMTWLQWDKEISNYTQIIYGLLEESQNQQEKNEQDLL
```

```
                   SOSIP.664 truncation
BG505.T332N     ALDKWASLWNWFDISNWLWYIKIFIMIVGGLIGLRIVFAVLSVIHRVRQGYSPLSFQTHT
MG505.A2        ALDKWASLWNWFDISNWLWYIKIFIMIVGGLIGLRIVFAMLSVIHRVRQGYSPLSFQTHT
MG505.H3        ALDKWASLWNWFDISNWLWYIKIFIMIVGGLIGLRIVFAVLSVIHRVRQGYSPLSFQTHT
```

Unknown
Formatted: Font:(Default) Courier, Bold, Font color: Light Blue

```
BG505.T332N     PNPRGLDRPERIEEEDGEQDRGRSTRLVSGFLALAWDDLRSLCLFCYHRLRDFILIAARI
MG505.A2        PNPRGLDRPERIEEEDGEQDRGRSTRLVSGFLALAWDDLRSLCFFCYHRLRDFILIAARI
MG505.H3        PNPRGLDRPERIEEEDGEQDRGRSTRLVSGFLALAWDDLRGLCLFSYHRLRDFILIAARI
```

```
BG505.T332N     VELLGHSSLKGLRLGWEGLKYLWNLLAYWGRELKISAINLFDTIAIAVAEWTDRVIEIGQ
MG505.A2        VELLGHSSLKGLRLGWEGLKYLWNLLAYWGRELKISAINLFDTIAIAVAGWTDRVIEIGQ
MG505.H3        VELLGHSSLKGLRLGWEGLKYLWNLLAYWGQELKISAINLFDTIAIAVAGWTDRVIEIGQ
```

```
BG505.T332N     RLCRAFLHIPRRIRQGLERALL
MG505.A2        RLCRAFLHIPRRIRQGFERALL
MG505.H3        RLCRAFLHIPRRIRQGFERALL
```

C. CZA97 cl.29 *vs.* cl.12

```
97.29          MRVRGIPRNWPQWWIWGILGFWMIIICRVVGNMWVTVYYGVPVWTDAKTTLFCASDAKAY
97.12          MRVRGIPRNWPQWWMWGILGFWMIIICRVVGNMWVTVYYGVPVWTDAKTTLFCASDTKAY
```

```
                                           88
97.29          DREVHNVWATHACVPTDPNPQEIVLENVTENFNMWKNDMVDQMHEDIISLWDQSLKPCVK
97.12          DREVHNVWATHACVPTDPNPQEIVLENVTENFNMWKNDMVDQMHEDIISLWDQSLKPCVK
```

```
                        130 133  139         156 160
97.29          LTPLCVTLHCTNATFKNNVTNDMNKEIRNCSFNTTTEIRDKKQQGYALFYRPDIVLLKEN
97.12          LTPLCVTLHCTNATFKNNVTNDMNKEIRNCSFNTTTEIRDKKQQGYALFYRPDIVLLKEN
```

```
                183 186      197                                 230        241
97.29           RNNSNNSEYILINCNASTITQACPKVNFDPIPIHYCAPAGYAILKCNNETFSGKGPCNNV
97.12           RNNSNNSEYILINCNASTITQACPKVNFDPIPIHYCAPAGYAILKCNNKTFSGKGPCNNV

                                 262           276          289        301
97.29           STVQCTHGIKPVVSTQLLLNGSLAEKEIIIRSENLTDNVKTIIVHLNKSVEIVCTRPNNN
97.12           STVQCTHGIKPVVSTQLLLNGSLAEKEIIIRSENLTDNVKTIIVHLNKSVEIVCTRPNNN

                                            332     339                355
97.29           TRKSMRIGPGQTFYATGDIIGDIRQAYCNISESMWKETLERVKEKLKENYNNNKTIKFAP
97.12           TRKSMRIGPGQTFYATGDIIGDIRQAYCNISGSKWNETLKRVKEKLQENYNNNKTIKFAP

                                      386      392 411
97.29           SSGGDLEITTHSFNCRGEFFYCNTSKLFSYNGTGNETIILPCRIKQIINMWQGVGRAMYA
97.12           SSGGDLEITTHSFNCRGEFFYCNTTRLFNNNATEDETITLPCRIKQIINMWQGVGRAMYA

                    442   448            462
97.29           PPIAGNITCKSNITGLLLVRDGGEDNKTEETFRPGGGNMKDNWRSELYKYKVIELKPLGI
97.12           PPIAGNITCKSNITGLLLVRDGGEDNKTEEIFRPGGGNMKDNWRSELYKYKVIELKPLGI

                               furin cleavage
97.29           APTGAKRRVVEREKRAVGIGAVFLGFLGAAGSTMGAASLALTVQARQLLSGIVQQQSNLL
97.12           APTGAKRRVVEREKRAVGIGAVFLGFLGAAGSTMGAASLTLTVQARQLLSSIVQQQSNLL

                                                                       611  616
97.29           RAIEAQQHMLQLTVWGIKQLQTRVLAIERYLKDQQLLGIWGCSGKLICTTNVPWNSSWSN
97.12           RAIEAQQHMLQLTVWGIKQLQTRVLAIERYLKDQQLLGIWGCSGKLICTTNVPWNSSWSN

                    625         637                           SOSIP.664 truncation
97.29           KSQTDIWNNMTWMEWDREISNYTDTIYRLLEDSQTQQEKNEKDLLALDSWKNLWNWFDIT
97.12           KSQTDIWNNMTWMEWDREISNYTDTIYRLLEDSQTQQEKNEKDLLALDSWKNLWSWFDIS

97.29           NWLWYIKIFIMIVGGLIGLRIIFAVLSIVNRVRQGYSPLSFQTLTPNPRGLDRLGRIEEE
97.12           NWLWYIKIFIMIVGGLIGLRIIFAVLSIVNRVRQGYSPLSFQTLTPNPRELDRLGRIEEE

97.29           GGEQDRDRSIRLVNGFLALAWDDLRSLCLFIYHQLRDFILLTARAVELLGRSSLRGLQRG
97.12           GGEQDRDRSIRLVNGFLALAWDDLRSLCLFIYHQLRDFILLTARAVELLGRSSLRGLQRG

97.29           WEALKYLGNLVQYWGLELKKSAISLLDTIAITVAEGTDRIIEVVQRICRAIRNIPRRIRQ
97.12           WEALKYLGNLVQYWGLELKKSAISLLDTIAITVAEGTDRIIEVVQRICRAIRNIPRRIRQ

97.29           GFEAALL
97.12           GFEAALP
```

Unknown
Formatted: Font:(Default) Courier, Bold, Font color: Light Blue

Ian Wilson 6/12/2016 7:43 AM
Comment [1]: Sorry cant seem to move over slightly to be between D and S – Help!!

Unknown
Formatted: Font:(Default) Courier, Bold, Font color: Light Blue

D. Heterologous Tier-2 viruses

```
Ce703010217_B6      MRVTGTQRNYPRWWIWGWWIWGILGFWMLLNCNG--AKDMWVTVYYGVPVWREAKTTLFC
246_F3_C10_2        MRARGMLRTW-----QHWWIWGILGFWMLMICN---MQDLWVTVYYGVPVWKDAKTTLFC
CNE55x2             MRVKETQMNW-----PNLWKWGVLILGLVIICSA--SDKLWVTVYYGVPVWRDADTTLFC
TRO11x3             MRAKGIRKNC-----QHLWIWGTMLLGMLMIYSAAEQGQLWVTVYYGVPVWKDASTTLFC
X1632_S2_B10        MKVKGTQRDW-----HSLWNWGILILGLVIICSA--SNNLWVTVYYGVPVWEDADTTLFC
BJOX002000032       MRVTGIRKNY-----WHLWRWGTMLLGMLMICST--VGNLWVTVYYGVPVWKEATTTLFC
CH11910             MRVTGIRKNY-----RHLWRWGTMLLGMLMICSA--VGNLWVTVYYGVPVWKEATTTLFC
25710_243           MRVRGTLRNY-----QQWWIWGVLGFWMLMICNV--GGNLWVTVYYGVPVWKEATTTLFC
Ce1176_A3           MRAKGILRNY-----QQWWIWGILGFWMLMVCNV--VGNLWVTVYYGVPVWKEAKTTLFC


                                                   88
Ce703010217_B6      ASDAKAYEREVHNVWATHACVPTDPNPQERVLENVTENFNMWKNNMVDQMHEDIISLWDE
246_F3_C10_2        ASDAKAYEKEVHNVWATHACVPTDPNPQEIVMANVTEEFNMWKNNMVEQMHTDIISLWDQ
CNE55x2             ASDAKAHETEVHNVWATHACVPTDPNPQEIHLVNVTENFNMWKNKMVEQMQEDVISLWDE
TRO11x3             ASDAKAYDTEVHNVWATHACVPTDPNPQEVVLGNVTENFNMWKNNMVDQMHEDIISLWDQ
X1632_S2_B10        ASDAKAYSTESHNVWATHACVPTDPNPQEIYLENVTEDFNMWENNMVEQMQEDIISLWDE
BJOX002000032       ASDAKAYDTEVHNVWATHACVPTDPDPQEMFLENVTENFNMWKNNMVDQMHEDVISLWDQ
CH11910             ASDAKAYDTEVHNVWATHACVPTDPSPQELVLENVTENFNMWKNEMVNQMHEDVISLWDQ
25710_243           ASDAKAYDKEVHNVWATHACVPTDPNPQEMVLGNVTENFNMWKNEMVNQMHEDVISLWDQ
Ce1176_A3           ASDAKAYEKEVHNVWATHACVPTDPNPQEMVLENVTENFNMWKNDMVDQMHEDVISLWDQ


                                   130                                156 160
Ce703010217_B6      SLKPCIKLTPLCVTLNCGNAIV--N---------------ESTIEGMKNCSFNVTTELKD
246_F3_C10_2        SLKPCVKLTPLCVTLDCKDYNY--S-ITNNS---------TGMEGEIKNCSYNITTELRD
CNE55x2             SLKPCVKLTPLCVTLNCTTAN--TNETKNNTT-------DDNIKDEMKNCTFNMTTEIRD
TRO11x3             SLKPCVKLTPLCVTLNCTDNIT--NTNTNSSKNSSTHSYNNSLEGEMKNCSFNITAGIRD
X1632_S2_B10        SLKPCVKLTPLCVTLTCTNVTNVTDSVGTNSR-------LKGYKEELKNCSFNTTTEIRD
BJOX002000032       SLKPCVKLTPLCVTLECKNVNS--SS-SDTK---------NGTDPEMKNCSFNATTELRD
CH11910             SLKPCVKLTPLCVTLECSKVSN--NE-TDKY---------NGTE-EMKNCSFNATTVVRD
25710_243           SLKPCVKLTPLCVTLECSNVT---------Y---------NESMKEVKNCSFNLTTELRD
Ce1176_A3           SLKPCVKLTPLCVTLTCTNTTV--SNGSSNS---------NANFEEMKNCSFNATTEIKD


                                                         197
Ce703010217_B6      KKKKEYALFYKLDVVPLNGENNNSNSKNFSEYRLINCNTSTITQACPKVSFDPIPIHYCA
246_F3_C10_2        KRQKVYSLFYRLDVVQINDSNDRNNSQ----YRLINCNTTTMTQACPKVTFDPIPIHYCA
CNE55x2             KKQRVSALFYKLDIVPIDDSKNNSE------YRLINCNTSVIKQACPKVSFDPIPIHYCT
TRO11x3             KVKKEYALFYKLDVVPIEEDKDTNKTT----YRLRSCNTSVITQACPKVTFEPIPIHYCA
X1632_S2_B10        KKKQEYALFYKLDIVPINDNSNNSNG-----YRLINCNVSTIKQACPKVSFDPIPIHYCA
BJOX002000032       RKQKVYALFYKLDIVPLNEKNSSE-------YRLINCNTSTITQACPKVTFDPIPIHYCT
CH11910             RQQKVYALFYRLDIVPLTEKNSSENSS--KYYRLINCNTSAITQACPKVSFEPIPIHYCT
25710_243           KKQKVHALFYRLDIVPLNDTEKKN-SS--RPYRLINCNTSAITQACPKVTFDPIPIHYCT
Ce1176_A3           KKKNEYALFYKLDIVPLNNSSGKY-------R-LINCNTSAIAQACPKVTFEPIPIHYCA


                                230 234    241                262          276
Ce703010217_B6      PAGFAILKCNNETFNGTGPCNNVSTVQCTHGIKPVVSTQLLLNGSLAEKEIIIRSENLTN
246_F3_C10_2        PAGFAILKCNNKTFNGKGPCNNVSSVQCTHGIKPVVSTQLLLNGSLAEKEIVIRSENLTD
CNE55x2             PAGYVILKCNDKNFNGTGPCKNVSSVQCTHGIKPVVSTQLLLNGSLAEEEIIIRSENLTD
TRO11x3             PAGFAILKCNDKKFNGTGPCTNVSTVQCTHGIRPVVSTQLLLNGSLAEEEVVIRSENFTN
X1632_S2_B10        PAGFAILKCRDKEFNGTGTCRNVSTVQCTHGIKPVVSTQLLLNGSLAEGDIVIRSENITD
BJOX002000032       PAGYAILKCNDEKFNGTGPCSNVSTVQCTHGIKPVVSTQLLLNGSLAEKGIVIRSENLTN
CH11910             PAGYAILKCNDKTFNGTGPCHNVSTVQCTHGIKPVVSTQLLLNGSLAEGEIIIRSENLTN
25710_243           PAGYAILKCNDKKFNGTGPCHKVSTVQCTHGIKPVVSTQLLLNGSLAEGEIIIRSENLTN
Ce1176_A3           PAGYAILKCNNKTFNGTGPCNNVSTVQCTHGIKPVVSTQLLLNGSLAEKEIIIRSENLTN


                               289   295   301                            332   339
Ce703010217_B6      NAKIIIVHLNNPVKIICTRPGNNTRKSMRIGPGQTFYATGDIIGDIRRAYCNISEKTWYD
246_F3_C10_2        NVKTIIVHLNESVEINCTRPNNNTRKSVRIGPGQTFYATGDIIGNIRQAHCTVNKTEWNT
```

```
CNE55x2                 NAKNIIVHLNKSVEINCTRPSNNTRTSVRIGPGQVFYRTGDITGDIRKAYCEIDGTEWNK
TRO11x3                 NAKTIIVQLNESIAINCTRPNNNTRRSIHIGPGRAFYATGDIIGDIRQAHCNISRTEWNS
X1632_S2_B10            NAKTIIVHLNKTVSITCTRPNNNTRKSIRIGPGQALYATGAIIGDTRQAHCNINGSEWYE
BJ0X002000032           NVKTIIVHLNQSVEILCIRPNNNTRKSIRIGPGQTFYATGEIIGDIRQAHCNISGKVWNE
CH11910                 NVKTILVHLNQSVEIVCTRPNNNTRKSIRIGPGQTFYATGDIIGDIRQAHCNIS--KWHE
25710_243               NAKTIIVHLNQSVEIVCARPSNNTRTSIRIGPGQTFYATGAITGDIRQAHCNISKDKWNE
Ce1176_A3               NAKTIIIHFNESVGIVCTRPSNNTRKSIRIGPGQTFYATGDIIGDIRQAHCNVSKQNWNR

                                        355   363                    386   392
Ce703010217_B6          TLKNVSDKFQEHF--PNASIEFKPSAGGDLEITTHSFNCRGEFFYCDTSELFNGTYN---
246_F3_C10_2            ALTRVSKKLKEYF--PNKTIAFQPSSGGDLEITTFSFNCRGEFFYCNTSDLFNGTFN---
CNE55x2                 TLTQVAEKLKEHF---NKTIVYQPPSGGDLEITMHHFNCRGEFFYCNTTQLFNNS-----
TRO11x3                 TLRQIVTKLREQLGDPNKTIIFNQSSGGDTEITMHSFNCGGEFFYCNTTKLFNSTWN--G
X1632_S2_B10            MIQNVKNKLNETF---KKNITFNPSSGGDLEITTHSFNCRGEFFYCNTSELFNSSHL---
BJ0X002000032           TLQRVGEKLAEYF--PNKTIKFNSSSGGDLEITTHSFNCGGEFFYCNTSKLFNGTFN--G
CH11910                 TLKRVSEKLAEHF--PNKTINFTSSSGGDLEITTHSFTCRGEFFYCNTSGLFNSTYMPNG
25710_243               TLQRVGEKLAEHF--PNKTIKFNSSSGGDLEITTHSFNCRGEFFYCNTSGLFNGTFN--G
Ce1176_A3               TLQQVGRKLAEHF--PNRNITFNHSSGGDLEITTHSFNCRGEFFYCNTSGLFNGTYHPNG

                                      411                            442   448
Ce703010217_B6          --N-STYN---SSNNITLQCKIKQIINMWQGVGRAMYAPPIAGNITCESNITGLLLTRDG
246_F3_C10_2            -ETSGQFN---STFNSTLQCRIKQIINMWQEVGQAMYAPPIAGSITCISNITGLILTRDG
CNE55x2                 ----------VGNSTIKLPCRIKQIINMWQGVGQAMYAPPISGAINCLSNITGILLTRDG
TRO11x3                 NNTTESDS---TGENITLPCRIKQIINLWQEVGKAMYAPPIKGQISCSSNITGLLLTRDG
X1632_S2_B10            --FNGSTL--STNGTITLPCRIKQIVRMWQRVGQAMYAPPIAGNITCRSNITGLLLTRDG
BJ0X002000032           TYMPNVTE---GNSTISIPCRIKQIINMWQKVGRAMYAPPIEGNITCKSKITGLLLERDG
CH11910                 TYLHGDTN---SNSSITIPCRIKQIINMWQEVGRAMYAPPIEGNITCKSNITGLLLVRDG
25710_243               TYVSPNSTDSNSSSIITIPCRIKQIINMWQEVGRAMYAPPIAGNITCKSNITGLLLVRDG
Ce1176_A3               TYNETAVN---SSDTITLQCRIKQIINMWQEVGRAMYAPPIAGNITCNSTITGLLLTRDG

                               462                                furin cleavage
Ce703010217_B6          GNN-----KSTPETFRPGGGDMRDNWRSELYKYKVVEIKPLGIAPTKAKRRVVEREKRAV
246_F3_C10_2            GNT-----NSTKETFRPGGGNMRDNWRSELYKYKVVKIEPLGVAPTKARRRVVEREKRAV
CNE55x2                 GGNN---RSNE--TFRPGGGNIKDNWRSELYKYKVVEIEPLGIAPTKAKRRVVEREKRAV
TRO11x3                 GNNN----SSGPETFRPGGGNMKDNWRSELYKYKVIKIEPLGVAPTRAKRRVVQREKRAV
X1632_S2_B10            GTNK---DTNEAETFRPGGGDMRDNWRSELYKYKVVKIKPLGVAPTRARRRVVEREKRAI
BJ0X002000032           GPE------NDTEIFRPGGGDMRNNWRSELYKYKVVEIKPLGVAPTEAKRRVVERERRAV
CH11910                 GTESNNTETNNTEIFRPGGGDMRDNWRSELYKYKVVEIKPLGVAPTAAKRRVVEREKRAV
25710_243               GTGS---ESNKTEIFRPGGGDMRDNWRSELYKYKVVEIKPLGVAPTKAKRRVVEREKRAV
Ce1176_A3               GIN-----QTGEEIFRPGGGDMRDNWRNELYKYKVVEIKPLGIAPTKAKRRVVEREKRAV

Ce703010217_B6          G-MGAVFLGFLGAAGSTMGAASLTLTVQARQLLSGIVQQQNNLLRAIEAQQHMLQLTVWG
246_F3_C10_2            G-IGAVFIGFLGAAGSTMGAASITLTVQARQLLSGIVQQQSNLLRAIEAQQHLLKLTVWG
CNE55x2                 G-IGAMIFGFLGAAGSTMGAASITLTVQARQLLSGIVQQQSNLLRAIEAQQHMLQLTVWG
TRO11x3                 GTLGAMFLGFLGAAGSTMGAASVTLTVQARLLLSGIVQQQNNLLRAIEAQQHMLQLTVWG
X1632_S2_B10            G-LGTVLLGFLGTAGSTMGAASITLTVQVRQLLSGIVQQQSNLLRAIEAQQHLLQLTVWG
BJ0X002000032           G-IGAVFLGFLGVAGSTMGAASMALTVQARQLLSGIVQQQSNLLRAIEAQQHLLQLTVWG
CH11910                 G-IGAVFLGFLGVAGSTMGAASMTLTVQARQLLSGIVQQQSNLLRAIEAQQHLLQLTVWG
25710_243               G-IGAVFLGFLGAAGSTMGAASITLTVQARQLLSGIVQQQSNLLRAIEAQQHLLQLTVWG
Ce1176_A3               G-IGAVFLGFLGAAGSTMGAASITLTVQARQLLSGIVQQQSNLLRAIEAQQHMLQLTVWG

                                                              611  618       625
Ce703010217_B6          IKQLQARVLAIERYLTDQQLLGIWGCSGKLICTTNVPWNNSWSNKSYEDIWGRNMTWMQW
246_F3_C10_2            IKQLQARVLAVERYLKDQQLLGIWGCSGKLICTTNVPWNSSWSNKSQDEIWD-NMTWLQW
CNE55x2                 IKQLQARVLAVERYLKDQRFLGLWGCSGKTICTTAVPWNSTWSNKTYEEIWD-NMTWTQW
TRO11x3                 IKQLQARVLAVERYLRDQQLLGIWGCSGKLICTTNVPWNASWSNKSLNNIWE-NMTWMEW
X1632_S2_B10            IKQLQARVLAVERYLKDQQILGIWGCSGKLICTTNVPWNSSWSNKSYSDIWD-NLTWIQW
BJ0X002000032           IKQLQTRVLAIERYLKDQQLLGIWGCSGKLICTTAVPWNSSWSNKSQEEIWE-NMTWMQW
CH11910                 IKQLQTRVLAIERYLKDQQLLGIWGCSGKLICTTAVPWNSSWSNKSQKEIWD-NMTWMQW
```

Unknown
Formatted: Font:(Default) Courier, Bold, Font color: Light Blue

```
25710_243           IKQLQTRVLAIERYLKDQQLLGIWGCSGKLICTTAVPWNYSWSNRSQDDIWD-NMTWMQW
Ce1176_A3           IKQLQARVLAIERYLKDQQLLGIWGCSGKLICTTNVPWNSSWSNRSQEDIWN-NMTWMEW

                         637
Ce703010217_B6      DREINNYTNTIYRLLEKSQNQQEKNEKELLELDNWKNLWSWFNISNWLWYIKIFIMIVGG
246_F3_C10_2        DKEISNYTQIIYNLIEESQTQQELNERDLLALDKWANLWNWFDITKWLWYIKIFIMIVGG
CNE55x2             EREISNYTNQIYSILTESQSQQDKNEKDLLELDKWASLWNWFTLSRWLWYIKIFIMIVGG
TRO11x3             EREIDNYTDLIYILLEKSQIQQEKNEQELLELDSWASLWNWFDISKWLWYIKIFIMIVGG
X1632_S2_B10        EREISNYTQQIYTLLEESQNQQEKNEQELLALDKWASLWNWFDITNWLWYIKIFIMIVGG
BJ0X002000032       DKEISNYTDTIYRLLEDSQNQQERNEKDLLALDSWKNLWSWFDITNWLWYIRIFIMIVGG
CH11910             DKEISNYTNTIYKLLEDSQNQQESNEKDLLALDSWNNLWNWFNITQWLWYIKIFIIIVGG
25710_243           DKEISNYTNTIYKLLEDSQIQQEKNEKDLLALDSWENLWNWFNITNWLWYIKIFIIIVGG
Ce1176_A3           EREIDNYTHTIYSLLEESQIQQEKNEKDLLALDSWQNLWSWFSITKWLWYIKIFIMIVGG

Ce703010217_B6      LIGLRIIFAVLSIVNRVRQGYSPLSFQTLLPNPRG-PDRLGGIEEEGGEQDRDRSVRLVN
246_F3_C10_2        LIGLRIIFAVLSIVNRVRQGYSPLSFQTLTPNPRG-PDRPGGIEEEGGEQGRNSYTRLVS
CNE55x2             LIGLRIIFAVLSIVNRVRQGYSPLSFQTPILQQRE-PDRPERIEEGGGEQGRDTSVRFVS
TRO11x3             LVGLRIVFAVLSIANRVRQGYSPLSFQTRLPTPRG-PDRPEGIEKEGGGRDRDGSRPLVH
X1632_S2_B10        LIGLRIVFAVLSIINRVRKGYSPLSFQTLTRHQRE-PDRPGGIEEEDGEQDRDKSVRFVS
BJ0X002000032       LIGLRIIFAVLSIVNRVRQGYSPLSFQTLTPNPGG-PDRLGRIEEEGGKQDRDRSVRLVS
CH11910             LIGLRIIFAVLSIVNRVRQGYSPLSFQTLTPTSGGRPDRLERIEEEGGEQDRDRSIRLVN
25710_243           LIGLRIIFAVLPIVNRVRQGYSPLSFQTHTPTPGG-PDRLGRIEEEGGEQDNVRSIRLVN
Ce1176_A3           LIGLRIIFAVLSIVNRVRQGYSPLSLQTLIPNPRG-PDRLGRIEEEGGEQDRDRSIRLVN

Ce703010217_B6      GFLALAWDDLRNLCLFSYRQLRDFILIVARAVQLLGHSSLRGIQRGWEALKYLGSLVQYW
246_F3_C10_2        GFLPLAWDDLRSLCLFSYHLLRDFILIAARAAELLGRSSLRGLQRGWETLKYLGSLVQYW
CNE55x2             RFLALAWEDLRNLCLFSYHRLRDLLLIATRTVEILGHYSLKGLRRGWEVLKYLGNLLLYW
TRO11x3             GLLALIWDDLRSLCLFSYHRLRDLLLIVTRTVELLGR-------RGWELLKYWWNLLQYW
X1632_S2_B10        GFLSPVWDDLRSLCLFSYRRLRDFILVAARTVELLGRSSLKGLRLGWEGLKYLWNLLLYW
BJ0X002000032       GFLALAWDDLRNLCLFSYHRLRDFILVAVRVVELLGRNSLKGLQRGWEALKYLGSLVQYW
CH11910             GFLALAWDDLRNLCLFSYHRLRDFILVAARVVELLGRTSLRGLQRGWEALKYLGSLVQYW
25710_243           GFLALAWDDLRNLCLFSYHRLRDFILVAARVVELLGRNSLRGLQKGWEALKYLGSLVQYW
Ce1176_A3           GFLALAWDDLRSLCLFSYHQLRDFILVIARAVELLG-------QRGWEALKYLGSLVQYW

Ce703010217_B6      GLELKKSAISLLDTIAITVAEGTDRIIEVVQVIWRAILNIPRRIRQGFEAALQ
246_F3_C10_2        GLELKKSAISLLDTIAIQVAEGTDRIIELIQGIYRAIRNIPRRIRQGAETALV
CNE55x2             GQELKTSAISLLDATAIATAEWTDRVIEVAQRAWRAFIHIPRRIRQGFERALL
TRO11x3             SQELKNSAVSLLNTTAIAVAEGTDRVIEVVQRAFRAILHIPARIRQGLERALL
X1632_S2_B10        GRELKSSAINLLDTTAIAVANWTDRVIEVGQRIVRAFLHIPVRIRQGLERALL
BJ0X002000032       GQELKKSIISLVDTIAIAVAEGTDRIIELVQRFCRGIYHIPRRIRQGFEAALQ
CH11910             GQELKKSAISLVDTIAIVVAEGTDRIIDIVQAFCRAIYNIPRRIRQGFEAALQ
25710_243           GLELKRSAISLLDTIAIAVAEGTDRIIQLGQGICRAICNIPRRIRQGLEAALQ
Ce1176_A3           GIELKKSATSLLDTTAIVVVEGTDRIIGIIQAICRALLNLPRRIRQGFEAALL
```
