# Supplementary figures and images for: Sequential and Simultaneous Immunization of Rabbits with HIV-1 Envelope Glycoprotein SOSIP.664 Trimers from Clades A, B and C
Source: PLoS Pathog. 2016 Sep 14;12(9):e1005864. doi: 10.1371/journal.ppat.1005864 (PMC5023125; doi:10.1371/journal.ppat.1005864)

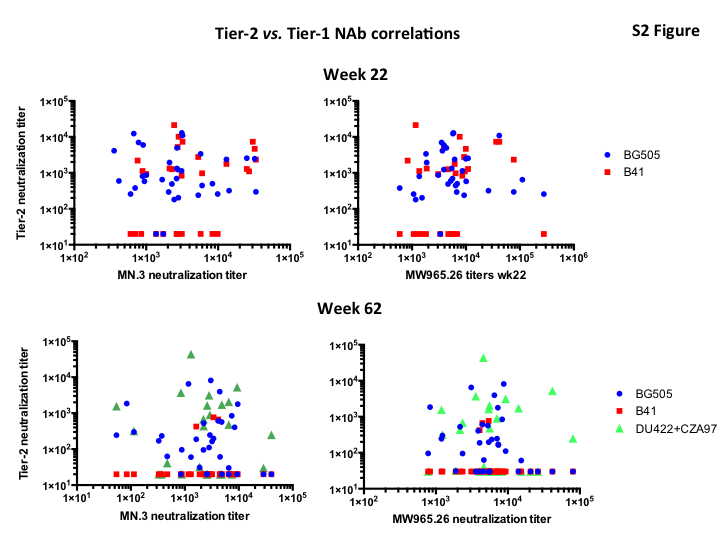

Supplement: S2 Fig — Top panels: NAb titers at week-22 are compared for the Tier-1 viruses MN.3 (left) and MW965.26 (right) and the autologous Tier-2 viruses BG505.T332N (excluding group 1 because of weak neutralizing responses) and B41 (excluding groups 3 and 6, which were negative for neutralization), as indicated. Bottom panels: NAb titers at week-62 are compared for the Tier-1 viruses MN.3 (left) and MW965.26 (right) and the autologous Tier-2 viruses BG505.T332N, B41, DU422 (group 1) and CZA97 (groups 2, 3, 4 and 8). The week-62 BG505.T3322N and B41 correlations with Tier-1 titers involve all the groups of rabbits bled at that time point, since some cross-neutralizing responses had arisen against BG505.T332N, albeit generally at low titers. No cross-neutralization of B41 was observed at week-62, however. Spearman-correlation analyses of these comparisons are recorded in S2 Table. (TIFF) [file ppat.1005864.s003.tiff]

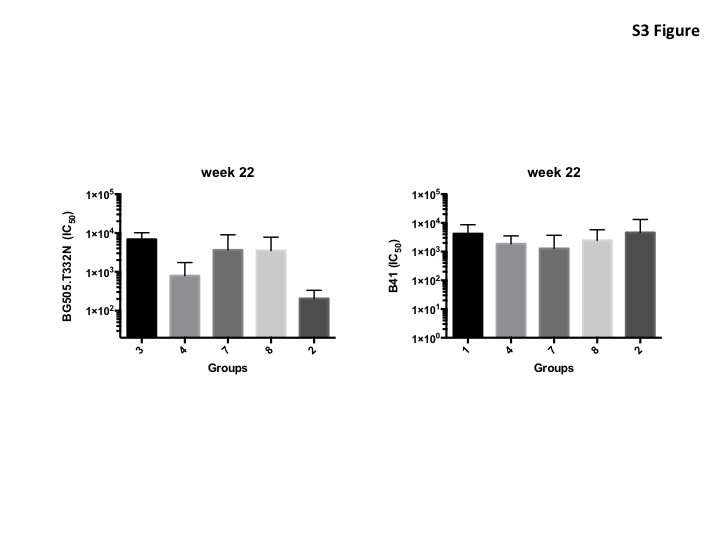

Supplement: S3 Fig — NAb titers against the BG505.T332N (left) and B41 (right) viruses are compared at the peak (week-22) of the responses to single or dual immunizations as follows: group 1 (30 μg clade B trimer), group 3 (30 μg clade A trimer), group 4 (30 μg total, 60% clade A and 40% clade B), group 7 (30 μg total, 40% clade A and 60% clade B), group 8 (90 μg total, 40% clade A and 60% clade B) and group-2 (30 μg total, 20% clade A and 80% clade B). The NAb titers (IC50, mean ± s.e.m. on a log-scale) are shown on the y-axis for the groups of five rabbits indicated on the category axis. (TIFF) [file ppat.1005864.s004.tiff]

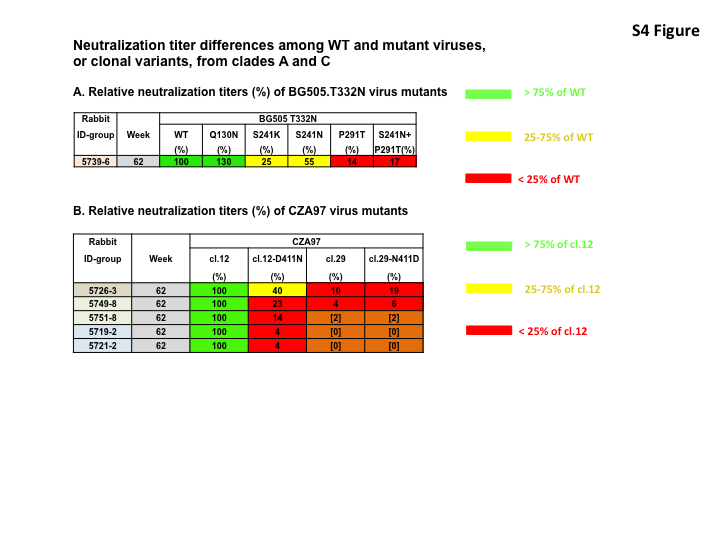

Supplement: S4 Fig — The values shown represent the reductions in neutralization titer for (A) BG505.T332N virus mutants relative to wild-type (WT, = 100%); or (B) CZA97 clones or mutants thereof relative to cl.12 (= 100%). The sera listed are the subset from the groups presented in Fig 7 for which titration curves revealed significant reductions in neutralization sensitivity of various mutants or clones that were not apparent at a single serum dilution of 1/50 (BG505.T332N) or 1/60 (CZA97). The numbers in brackets in orange cells represent relative titers of sera that neutralized the respective viruses to an extent of <25% of WT (Fig 7); in those cases the titration was not necessary to show a neutralization difference. (TIFF) [file ppat.1005864.s005.tiff]

## Slide 1
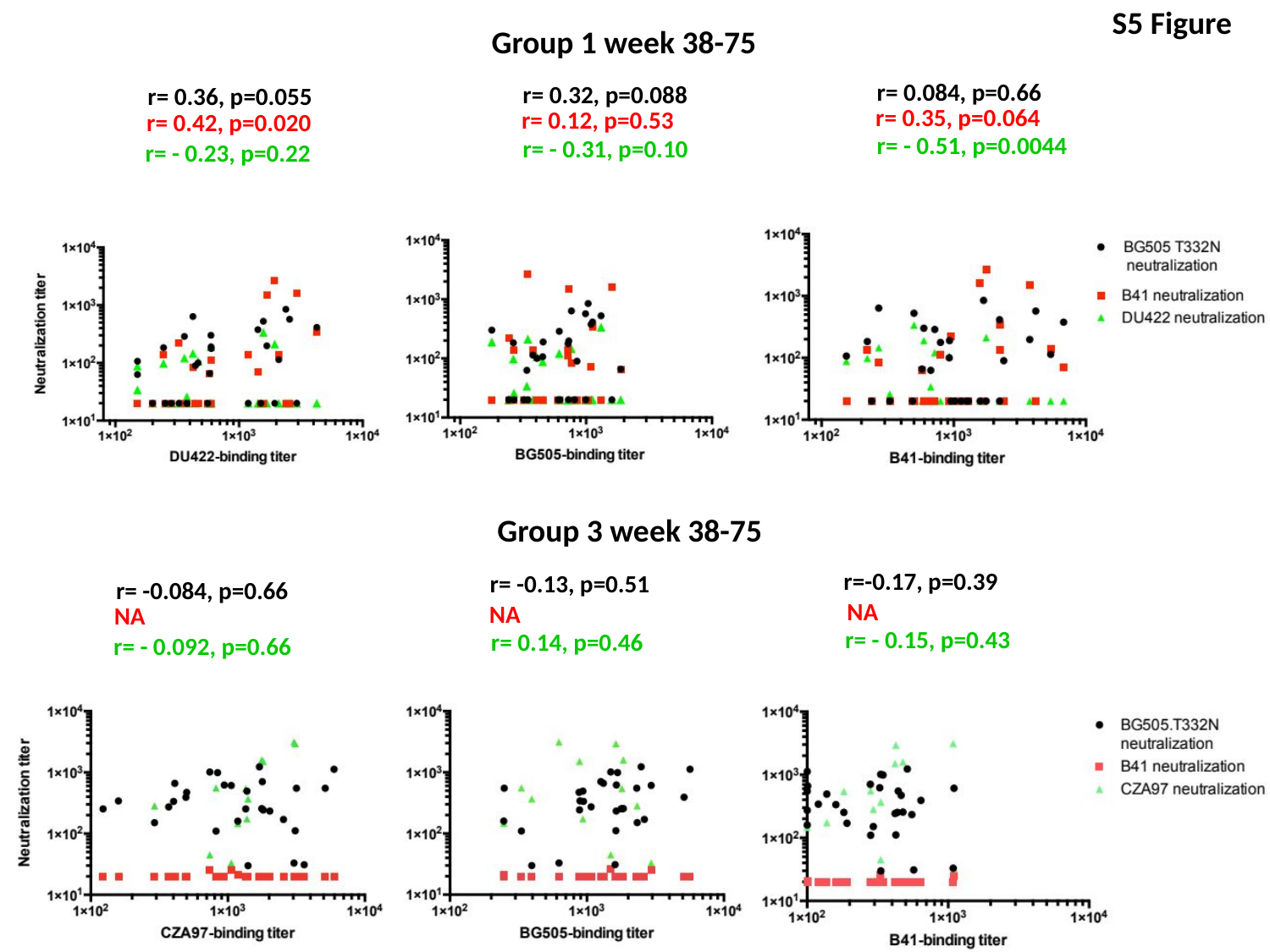

S5 Figure
Group 1 week 38-75
r= 0.084, p=0.66
r= 0.32, p=0.088
r= 0.36, p=0.055
r= 0.35, p=0.064
r= 0.12, p=0.53
r= 0.42, p=0.020
r= - 0.51, p=0.0044
r= - 0.31, p=0.10
r= - 0.23, p=0.22
Group 3 week 38-75
r=-0.17, p=0.39
r= -0.13, p=0.51
r= -0.084, p=0.66
NA
NA
NA
r= - 0.15, p=0.43
r= 0.14, p=0.46
r= - 0.092, p=0.66

Supplement: S5 Fig — The scatterplots show NAb titers on the y-axes and the antibody binding titers to SOSIP.664-D7324 trimers on the x-axes. Within each plot the symbols corresponding to neutralization of the BG505.T332N, B41, DU422 and CZA97 viruses are color-coded as indicated on the figure panels. Spearman correlation coefficients (r-values) and the corresponding significances (p-values) are color-coded analogously. The scatter plots show the BG505, B41 and DU422 or CZA97 NAb and binding antibody titers for groups-1 and -3, during the period of DU422 or CZA97 trimer boosting immunizations from weeks 38–62 (top panel, group-1, DU422 boosting; lower panel, group-3, CZA97 boosting). (PPTX) [file ppat.1005864.s006.pptx]
