# Supplementary material for: Sequential and Simultaneous Immunization of Rabbits with HIV-1 Envelope Glycoprotein SOSIP.664 Trimers from Clades A, B and C
Source: PLoS Pathog. 2016 Sep 14;12(9):e1005864. doi: 10.1371/journal.ppat.1005864 (PMC5023125; doi:10.1371/journal.ppat.1005864)
Supplement: S1 Table — (DOC) [file ppat.1005864.s007.doc]

**S1 Table. A**ntibody responses to the D7324-epitope tag on the B41-D7324 trimer

| **Antibody** | **Endpoint** |
| --- | --- |
|  | **µg/ml** |
| **D7324*^a^*** | **0.010 *^d^*** |
| **HIVIG*^b^*** | **84** |
| **HIVIG + serum 5715-1** | **91** |
|  |  |
| **Rabbit ID-group***^c^* | **Serum dilution** |
| **5713-1** | **<20 *^d^*** |
| **5714-1** | **<20** |
| **5715-1** | **<20** |
| **5716-1** | **<20** |
| **5717-1** | **<20** |
| **5718-2** | **<20** |
| **5719-2** | **<20** |
| **5720-2** | **<20** |
| **5721-2** | **<20** |
| **5722-2** | **<20** |
| **5723-3** | **<20** |
| **5724-3** | **<20** |
| **5728-4** | **<20** |
| **5729-4** | **<20** |
| **5743-7** | **<20** |
| **5744-7** | **<20** |

***^a^*** D7324 is an affinity-purified sheep Ab to the C5 region of gp120 that corresponds to the peptide used in the ELISA.

***^b^*** HIVIG is a human IgG preparation from a pool of sera from HIV-1-infected individuals.

***^c^*** The rabbit serum samples tested were from week-26, after 4 immunizations with D7324-epitope-tagged proteins (groups 2, 4 and 7) or non-tagged trimers (groups 1 and 3).

***^d^*** The values recorded are EC_50_ in µg/ml for D7324 and HIVIG, or the serum dilutions that yield 50% binding.
