# Supplementary material for: Sequential and Simultaneous Immunization of Rabbits with HIV-1 Envelope Glycoprotein SOSIP.664 Trimers from Clades A, B and C
Source: PLoS Pathog. 2016 Sep 14;12(9):e1005864. doi: 10.1371/journal.ppat.1005864 (PMC5023125; doi:10.1371/journal.ppat.1005864)
Supplement: S2 Table — (DOC) [file ppat.1005864.s008.doc]

**Table S2. Correlation coefficients for comparisons between Tier-1 and autologous Tier-2 NAb titers**

| **Time point** | **Comparison** | **r-value*^a^*** | **p-value*^a^*** |
| --- | --- | --- | --- |
| Week-22 | MN.3 *vs.* BG505.T332N | -0.034 | 0.84 |
|  | MN.3 *vs.* B41 | 0.32 | 0.082 |
|  | MW965.26 *vs.* BG505.T332N | 0.039 | 0.83 |
|  | MW965.26 *vs*. B41 | 0.31 | 0.097 |
|  |  |  |  |
| Week-62 | MN.3 *vs.* BG505.T332N | -0.16 | 0.36 |
|  | MN.3 *vs.* B41 | 0.026 | 0.88 |
|  | MN.3 *vs.* DU422 + CZA97 | 0.044 | 0.83 |
|  | MW965.26 *vs.* BG505.T332N | 0.27 | 0.12 |
|  | MW965.26 *vs*. B41 | -0.11 | 0.54 |
|  | MW965.26 *vs.* DU422 + CZA97 | -0.072 | 0.74 |

*^a^* The analyses are based on the same data as were used to derive the plots presented in Fig. S1. The relationships between the NAb titers were analyzed as Spearman correlations, yielding the correlation coefficients recorded in the table as r-values, with significances listed as p-values for two-tailed tests. The DU422 data are derived from group 1, CZA97 from groups 2, 4 and 8.
