# Supplementary material for: Sequential and Simultaneous Immunization of Rabbits with HIV-1 Envelope Glycoprotein SOSIP.664 Trimers from Clades A, B and C
Source: PLoS Pathog. 2016 Sep 14;12(9):e1005864. doi: 10.1371/journal.ppat.1005864 (PMC5023125; doi:10.1371/journal.ppat.1005864)
Supplement: S3 Table — (DOC) [file ppat.1005864.s009.doc]

**Table S3. Mapping of autologous Tier-2 NAb responses**

**A. Neutralization of BG505.T332N virus mutants**

| **Rabbit**  **ID-group** | **Week** | **BG505-Q130N**  **(%) ^a^** | **BG505-S241N**  **(%)** | **BG505-S241K**  **(%)** | **BG505-P291T**  **(%)** | **BG505-S241N+P291T**  **(%)** | **MG505 cl.A2**  **(%)** | **MG505**  **cl.A2 K241S**  **(%)** | **MG505 cl.H3**  **(%)** |
| --- | --- | --- | --- | --- | --- | --- | --- | --- | --- |
| 5713-1 | 38 | 110 ± 3 | **<10** | **<10** | **<10** | **<10** | **10 ± 16** | 110 ± 8 | **<10** |
| 5713-1 | 50 | 94 ± 1 | **<10** | **<10** | **<10** | **<10** | **<10** | 110 ± 2 | **<10** |
| 5713-1 | 62 | 95 ± 8 | **25 ± 11** | 39 ± 13 | 28 ± 25 | **20 ± 19** | **19 ± 2** | 84 ± 25 | **<10** |
|  |  |  |  |  |  |  |  |  |  |
| 5715-1 | 38 | 120 ± 1 | 85 ± 16 | 100 ± 1 | 65 ± 1 | 56 ± 2 | **<10** | **18 ± 1** | **<10** |
| 5715-1 | 62 | 100 ± 2 | 99 ± 9 | 100 ± 0 | 93 ± 5 | 97 ± 6 | **<10** | 29 ± 12 | **<10** |
|  |  |  |  |  |  |  |  |  |  |
| 5716-1 | 62 | 110 ± 5 | **<10** | **<10** | **<10** | **<10** | **<10** | 40 ± 51 | **<10** |
|  |  |  |  |  |  |  |  |  |  |
| 5717-1 | 62 | 73 ± 16 | **10 ± 14** | **6 ± 6** | **<10** | **<10** | **<10** | 120 ± 9 | **<10** |
|  |  |  |  |  |  |  |  |  |  |
| 5723-3 | 22 | 100 ± 0 | 100 ± 2 | 100 ± 2 | 87 ± 2 | 82 ± 2 | **<10** | 79 ± 9 | **<10** |
| 5723-3 | 38 | 100 ± 4 | 70 ± 4 | 70 ± 5 | 46 ± 3 | 40 ± 6 | **<10** | 94 ± 4 | **<10** |
|  |  |  |  |  |  |  |  |  |  |
| 5724-3 | 22 | 100 ± 1 | 99 ± 1 | 100 ± 1 | 100 ± 0 | 98± 1 | **12 ± 2** | **<10** | **<10** |
| 5724-3 | 26 | 100 ± 0 | 100 ± 0 | 100 ± 1 | 99 ± 0 | 100 ± 0 | **<10** | **15 ± 5** | **<10** |
| 5724-3 | 62 | 100 ± 1 | 94 ± 2 | 100 ± 4 | 84 ± 10 | 71 ± 13 | **<10** | **<10** | **<10** |
|  |  |  |  |  |  |  |  |  |  |
| 5725-3 | 22 | 100 ± 7 | **<10** | **17 ± 6** | **<10** | **<10** | **<10** | 99 **±** 0 | **<10** |
|  |  |  |  |  |  |  |  |  |  |
| 5726-3 | 22 | 97 ± 3 | **<10** | **<10** | **<10** | **<10** | **<10** | 100 ± 1 | **<10** |
| 5726-3 | 38 | 100 ± 1 | 13 ± 32 | **<10** | **<10** | **<10** | 34 ± 27 | 84 ± 13 | **<10** |
|  |  |  |  |  |  |  |  |  |  |
| 5727-3 | 22 | 98 ± 2 | 110 ± 2 | 110 ± 1 | 100 ± 1 | 100 ± 2 | **<10** | 46 ± 2 | **<10** |
| 5727-3 | 50 | 100 ± 1 | 71 ± 20 | 78 ± 20 | 43 ± 19 | 56 ± 11 | **<10** | 67 ± 5 | **<10** |
| 5727-3 | 62 | 100 ± 4 | 57 ± 11 | 72 ± 12 | 42 ± 9 | 49 ± 15 | **<10** | **21 ± 43** | **<10** |
|  |  |  |  |  |  |  |  |  |  |
| 5729-4 | 22 | 110 ± 5 | **<10** | **<10** | **<10** | **<10** | **<10** | 53 ± 23 | **<10** |
|  |  |  |  |  |  |  |  |  |  |
| 5730-4 | 22 | 97 ± 1 | 47 ± 24 | 83 ± 8 | **25 ± 11** | 44 ± 10 | **<10** | 64 ± 12 | **<10** |
| 5730-4 | 38 | 95 ± 6 | 77 ± 6 | 68 ± 11 | 51 ± 13 | 31 ± 3 | **<10** | 50 ± 16 | **<10** |
|  |  |  |  |  |  |  |  |  |  |
| 5731-4 | 22 | 100 ± 2 | 97 ± 4 | 110 ± 4 | 86 ± 5 | 90 ± 4 | **<10** | **<10** | **<10** |
|  |  |  |  |  |  |  |  |  |  |
| 5743-7 | 22 | 100 ± 1 | 99 ± 1 | 100 ± 1 | 99 ± 1 | 98 ± 1 | 100 ± 0 | 100 ± 1 | **<10** |
|  |  |  |  |  |  |  |  |  |  |
| 5744-7 | 26 | 100 ± 0 | 97 ± 0 | 99 ± 0 | 97 ± 1 | 96 ± 3 | 100 ± 0 | 100 ± 1 | **<10** |
|  |  |  |  |  |  |  |  |  |  |
| 5746-7 | 22 | 110 ± 10 | 110 ± 9 | 100 ± 3 | 86 ± 12 | 84 ± 9 | **11 ± 35** | 93 ± 1 | **<10** |
|  |  |  |  |  |  |  |  |  |  |
| 5747-7 | 22 | 100 ± 5 | **<10** | **<10** | **<10** | **<10** | **<10** | 84 ± 6 | **<10** |
|  |  |  |  |  |  |  |  |  |  |
| 5748-8 | 22 | 100 ± 7 | 88 ± 14 | 78 ± 15 | 65 ± 12 | 37 ± 4.1 | **<10** | 86 ± 1 | **<10** |
| 5748-8 | 38 | 110 ± 7 | 50 ± 18 | 45 ± 11 | **<10** | **<10** | **<10** | 64 ± 28 | **<10** |
|  |  |  |  |  |  |  |  |  |  |
| 5749-8 | 22 | 98 ± 5 | **<10** | **<10** | **<10** | **<10** | **<10** | 100 ± 1 | **<10** |
| 5749-8 | 38 | 120 ± 12 | **<10** | **24 ± 22** | 28 ± 15 | **<10** | 29 ± 10 | 83 ± 41 | **<10** |
|  |  |  |  |  |  |  |  |  |  |
| 5750-8 | 22 | 100 ± 1 | 26 ± 22 | **17 ± 16** | 29 ± 27 | **<10** | **24 ± 25** | 100 ± 1 | **<10** |
| 5750-8 | 50 | 92 ± 24 | **<10** | **<10** | **<10** | **<10** | **<10** | 110 ± 4 | **<10** |
|  |  |  |  |  |  |  |  |  |  |
| 5751-8 | 22 | 100 ± 2 | 100 ± 1 | 100 ± 2 | 97 ± 3 | 98 ± 1 | **<10** | 88 ± 6 | **<10** |
| 5751-8 | 50 | 100 ± 14 | 67 ± 11 | 85 ± 11 | 72 ± 13 | 45 ± 1 | **<10** | 38 ± 12 | **<10** |
|  |  |  |  |  |  |  |  |  |  |
| 5752-8 | 22 | 110 ± 8.3 | **<10** | **<10** | **<10** | **<10** | **<10** | 62 ± 3 | **<10** |
|  |  |  |  |  |  |  |  |  |  |
| 5733-5 | 38 | 110 ± 4 | <10 | 20 ± 17 | <10 | <10 | 25 ± 11 | 69 ± 16 | <10 |
| 5733-5 | 62 | 73 ± 26 | 29 ± 38 | 34 ± 28 | 40 ± 1 | <10 | <10 | 17 ± 34 | <10 |
|  |  |  |  |  |  |  |  |  |  |
| 5734-5 | 22 | 94 ± 1 | <10 | <10 | <10 | <10 | <10 | 100 ± 6 | <10 |
| 5734-5 | 38 | 97 ± 4 | 42 ± 9 | 33 ± 13 | <10 | <10 | 37 ± 2 | 100 ± 6 | <10 |
| 5734-5 | 50 | 75 ± 2 | 44 ± 18 | 59 ± 6 | 25 ± 8 | 12 ± 15 | 39 ± 6 | 96 ± 3 | <10 |
| 5734-5 | 62 | 82 ± 15 | 47 ± 5 | 47 ± 22 | 41 ± 3 | 10 ± 14 | 48 ± 4 | 110 ± 14 | <10 |
|  |  |  |  |  |  |  |  |  |  |
| 5735-5 | 22 | 98 ± 6 | <10 | 47 ± 21 | <10 | 14 ± 20 | 25 ± 14 | 61 ± 2 | <10 |
| 5735-5 | 38 | 110 ± 16 | 47 ± 38 | <10 | <10 | <10 | 31 ± 3 | 27 ± 40 | <10 |
| 5735-5 | 62 | 110 ± 9 | 18 ± 40 | 60 ± 8 | <10 | <10 | 57 ± 4 | 57 ± 28 | <10 |
|  |  |  |  |  |  |  |  |  |  |
| 5736-5 | 22 | 92 ± 7 | <10 | <10 | <10 | <10 | <10 | 74 ± 22 | <10 |
|  |  |  |  |  |  |  |  |  |  |
| 5738-6 | 22 | 100 ± 3 | 82 ± 4 | 98 ± 4 | 84 ± 8 | 66 ± 5 | <10 | 47 ± 17 | <10 |
| 5738-6 | 38 | 100 ± 7 | 67 ± 29 | 110 ± 4 | 56 ± 26 | 74 ± 2 | <10 | 45 ± 13 | <10 |
| 5738-6 | 50 | 100 ± 2 | 100 ± 1 | 100 ± 1 | 97 ± 1 | 95 ± 1 | 18 ± 8 | 78 ± 7 | <10 |
| 5738-6 | 62 | 100 ± 1 | 100 ± 1 | 100 ± 1 | 98 ± 3 | 99 ± 0 | <10 | 83 ± 5 | <10 |
|  |  |  |  |  |  |  |  |  |  |
| 5739-6 | 22 | 99 ± 10 | <10 | 56 ± 24 | <10 | <10 | 98 ± 9 | 120 ± 9 | <10 |
| 5739-6 | 38 | 110 ± 12 | 56 ± 29 | 90 ± 4 | 54 ± 16 | <10 | 90 ± 6 | 100 ± 20 | <10 |
| 5739-6 | 50 | 99 ± 2 | 62 ± 27 | 69 ± 2 | 55 ± 13 | 12 ± 19 | 69 ± 2 | 94 ± 4 | <10 |
| 5739-6 | 62 | 100 ± 1 | 100 ± 2 | 98 ± 1 | 99 ± 3 | 90 ± 2 | 64 ± 5 | 93 ± 4 | <10 |
|  |  |  |  |  |  |  |  |  |  |
| 5740-6 | 22 | 100 ± 1 | 98 ± 2 | 100 ± 1 | 97 ± 1 | 94 | <10 | <10 | <10 |
| 5740-6 | 38 | 100 ± 1 | 100 ± 3 | 100 ± 2 | 96 ± 1 | 93 ± 2 | <10 | <10 | <10 |
| 5740-6 | 50 | 100 ± 1 | 99 ± 1 | 100 ± 1 | 98 ± 1 | 98 ± 1 | <10 | <10 | <10 |
| 5740-6 | 62 | 100 ± 0 | 100 ± 0 | 100 ± 0 | 100 ± 0 | 99 ± 1 | <10 | 62 ± 8 | 14 ± 10 |
|  |  |  |  |  |  |  |  |  |  |
| 5741-6 | 22 | 100 ± 1 | 100 ± 3 | 99 ± 1 | 89 ± 5 | 85 ± 4 | <10 | 6 ± 9 | <10 |
| 5741-6 | 38 | 100 ± 1 | 98 ± 1 | 98 ± 1 | 92 ± 2 | 93 ± 1 | <10 | <10 | <10 |
| 5741-6 | 50 | 100 ± 0 | 100 ± 0 | 100 ± 0 | 100 ± 1 | 100 ± 1 | 28 ± 7 | 39± 7 | 40 ± 5 |
| 5741-6 | 62 | 100 ± 0 | 100 ± 0 | 100 ± 1 | 100 ± 0 | 100 ± 1 | 16 ± 2 | 26± 9 | <10 |
|  |  |  |  |  |  |  |  |  |  |
| 5742-6 | 38 | 110 ± 2 | 100 ± 2 | 110 ± 1 | 94 ± 3 | 80 ± 5 | <10 | 26 ± 1 | <10 |
| 5742-6 | 50 | 100 ± 0 | 100 ± 0 | 100 ± 0 | 99 ± 1 | 100 ± 0 | <10 | 45 ± 1 | <10 |
| 5742-6 | 62 | 100 ± 1 | 100 ± 1 | 100 ± 1 | 99 ± 1 | 99 ± 1 | <10 | 45 ± 1 | <10 |
|  |  |  |  |  |  |  |  |  |  |
| CH01 | - | 83 ± 10 | 87 ± 5 | 100 | 100 ± 2 | 94 | 85 ± 2 | 85 | 95 ± 1 |
| VRC01 | - | 100 ± 1 | 99 ± 1 | 100 | 100 ± 1 | 100 | 100 ± 0 | 100 | 100 ± 1 |

*^a^* The values recorded for the various mutant viruses are the percentage neutralization at a dilution of 1/50, relative to the BG505.T332N parental virus (defined as 100%), and are the averages of 2 replicates ± s.e.m. Entries highlighted in bold indicate fully or substantially resistant viruses (≤ 25% neutralization). The Q130N, S241N and P291T changes introduce N-linked glycans at position 130, 241 and 289, respectively. The S241N+P291T double mutant contains glycans at both positions 241 and 289. The MG505 cl.A2 and cl.H3 viruses differ from BG505.T332N at several positions (see Fig. S1). Of note is that at position-241 MG505 cl.A2 has a lysine residue (i.e., as per the S241K mutant) whereas cl.H3 has a glycan site (i.e., as per the S241N mutant). A glycan is present at position-289 in both MG505 clones. The K241S change in MG505 cl.A2 restores the Ser residue that is present at position-241 in the BG505.T332N virus. The CH01 and VRC01 bNAbs were used as control reagents for assessing the overall neutralization sensitivity of the various viruses.

**B. Neutralization of B41 virus mutants**

| **Rabbit**  **ID-group** | **Week** | **B41-N132T**  **(%)*** | **B41-A291T**  **(%)** |
| --- | --- | --- | --- |
| 5713-1 | 22 | 110 ± 22 | 44 ± 14 |
| 5713-1 | 38 | 96 ± 4 | **<10** |
|  |  |  |  |
| 5714-1 | 22 | 120 ± 29 | **<10** |
| 5714-1 | 38 | 83 ± 11 | **<10** |
|  |  |  |  |
| 5716-1 | 22 | 120 ± 13 | **<10** |
| 5716-1 | 50 | 110 ± 6 | **<10** |
|  |  |  |  |
| 5717-1 | 22 | 110 ± 10 | **<10** |
| 5717-1 | 26 | 67 ± 8 | **<10** |
|  |  |  |  |
| 5718-2 | 38 | 120 ± 25 | **<10** |
|  |  |  |  |
| 5719-2 | 22 | 130 ± 28 | **<10** |
| 5719-2 | 26 | 110 ± 3 | **<10** |
| 5719-2 | 48 | 98 ± 5 | **<10** |
| 5719-2 | 50 | 120 ± 7 | **<10** |
|  |  |  |  |
| 5721-2 | 26 | 140 ± 2 | **<10** |
| 5721-2 | 38 | 58 ± 26 | **<10** |
|  |  |  |  |
| 5722-2 | 22 | 110 ± 4 | **<10** |
| 5722-2 | 26 | 120 ± 3 | **<10** |
| 5722-2 | 36 | 94 ± 24 | **<10** |
| 5722-2 | 38 | 99 ± 6 | **<10** |
|  |  |  |  |
| 5729-4 | 22 | 110 ± 2 | **<10** |
| 5729-4 | 24 | 96 ± 68 | **<10** |
| 5729-4 | 26 | 98 ± 27 | **<10** |
| 5729-4 | 38 | 130 ± 18 | **<10** |
|  |  |  |  |
| 5730-4 | 22 | 110 ± 12 | **25 ± 7** |
| 5730-4 | 24 | 93 ± 12 | 60 ± 1 |
| 5730-4 | 26 | 66 ± 25 | **<10** |
| 5730-4 | 38 | 110 ± 7 | 54 ± 22 |
|  |  |  |  |
| 5731-4 | 22 | 120 ± 11 | **<10** |
| 5731-4 | 24 | 140 ± 16 | **<10** |
|  |  |  |  |
| 5732-4 | 26 | 120 ± 15 | **<10** |
|  |  |  |  |
| 5734-5 | 50 | 110 ± 6 | **<10** |
| 5734-5 | 62 | 110 ± 8 | **<10** |
|  |  |  |  |
| 5735-5 | 22 | 100 ± 8 | **<10** |
| 5735-5 | 50 | 110 ± 10 | **<10** |
| 5735-5 | 62 | 100 ± 5 | **<10** |
|  |  |  |  |
| 5736-5 | 22 | 110 ± 8 | **<10** |
| 5736-5 | 26 | 97 ± 13 | **<10** |
| 5736-5 | 50 | 110 ± 1 | **<10** |
| 5736-5 | 62 | 98 ± 9 | **<10** |
|  |  |  |  |
| 5738-6 | 50 | 90 ± 9 | **<10** |
|  |  |  |  |
| 5739-6 | 38 | 100 ± 6 | **<10** |
|  |  |  |  |
| 5741-6 | 38 | 130 ± 5 | **<10** |
|  |  |  |  |
| 5746-7 | 22 | 130 ± 8 | **<10** |
| 5746-7 | 26 | 110 ± 2 | **<10** |
| 5747-7 | 22 | 110 ± 8 | **<10** |
|  |  |  |  |
| 5748-8 | 22 | 92 ± 10 | **<10** |
| 5748-8 | 26 | 110 ± 7 | **<10** |
|  |  |  |  |
| 5750-8 | 26 | 100 ± 1 | **<10** |
|  |  |  |  |
| CH01 | NA | 48 ± 27 | 120 ± 24 |
| VRC01 | NA | 98 ± 2 | 98 ± 3 |

*^a^* The values recorded for the various mutant viruses are the percentage neutralization at a dilution of 1/50, relative to the B41 parental virus (defined as 100%), and are the averages of two replicates ± s.e.m. Entries highlighted in bold indicate fully or substantially resistant viruses (≤ 25% neutralization). The N132T and A291T changes introduce N-linked glycans at position 132 and 289, respectively. The CH01 and VRC01 bNAbs were used as control reagents for assessing overall neutralization sensitivity.
